# Nine new species of *Itaplectops* (Diptera: Tachinidae) reared from caterpillars in Area de Conservación Guanacaste, northwestern Costa Rica, with a key to *Itaplectops* species

**DOI:** 10.3897/BDJ.3.e4596

**Published:** 2015-12-23

**Authors:** AJ Fleming, D. Monty Wood, M. Alex Smith, Daniel H. Janzen, Winnie Hallwachs

**Affiliations:** ‡ Agriculture Agri-Food Canada, Ottawa, Canada; § Department of Integrative Biology, Guelph, Canada; | University of Pennsylvania, Philadelphia, United States of America

**Keywords:** tropical rain forest, tropical dry forest, cloud forest, parasitoid fly, host-specificity, caterpillar, Uramyini

## Abstract

Nine new species of *Itaplectops* Townsend (Diptera: Tachinidae) are described from Area de Conservación Guanacaste (ACG), northwestern Costa Rica. All specimens have been reared from ­various species of ACG caterpillars in the families Limacodidae and Dalceridae. By combining morphological, photographic, and genetic barcode data we provide clear yet concise descriptions. The following nine new species are described in the genus *Itaplectops*: *Itaplectops akselpalolai*, *Itaplectops anikenpalolae*, *Itaplectops argentifrons*, *Itaplectops aurifrons*, *Itaplectops ericpalolai*, *Itaplectops griseobasis*, *Itaplectops omissus*, *Itaplectops shellymcsweeneyae*, *Itaplectops tristanpalolai*. We move *Itaplectops* to the tribe Uramyini from its original placement within the Blondeliini, and we discuss its systematic placement. We also provide a key differentiating the, genera of the tribe Uramyini as well as the known species of *Itaplectops*.

## Introduction

The tachinid genus *Itaplectops* Townsend is a small New World genus, previously known only from the type species (Guimarães 1971) and one previously unidentified species from Area de Conservación Guanacaste (ACG) in northwestern Costa Rica mentioned in Wood and Zumbado (2010) which is now described here. The genus was erected for a single female specimen obtained in Cantareira, Brasil, in 1927, described as *Itaplectops antennalis* Townsend, 1927. Here we describe nine new species from the genus *Itaplectops*, reared from wild caterpillars collected in ACG (Janzen et al. 2009).

Guimarães (1971) in his Neotropical catalog, placed *Itaplectops* Townsend in the tribe Blondeliini; however, according to the tribal concept set out by Wood (1985) the morphological characters of *Itaplectops* would exclude it from this tribe. Guimarães (1980) published the last major systematic work on the tribe Uramyini. In it he provided a diagnosis of the tribe and recognized only two valid genera: *Uramya* Robineau-Desvoidy 1830 and *Thelairaporia* Guimarães, 1980. We also discuss the inclusion of the genus *Itaplectops* Townsend in the tribe Uramyini.

All species described as new herein are based on differences in external morphology, male terminalia, and COI sequences (coxI or cytochrome oxidase 1, a.k.a. DNA barcodes). Our present study focuses on northwestern Costa Rica, and these nine new species are by no means considered by us to be representative of the whole *Itaplectops* diversity of the region.

## Materials and methods

### Abbreviations of depositories

CNC Canadian National Collection of Insects, Arachnids and Nematodes, Ottawa, Canada

USNM U.S. National Museum of Natural History, Washington, D.C., USA

INBio Instituto Nacional de Biodiversidad, Santo Domingo de Heredia, Costa Rica

### Geographic area of the study and rearing intensity

The flies described in this paper were collected as part of a 35+ year–old ongoing inventory of the caterpillars, their food plants and their parasitoids, found within the 125,000+ ha terrestrial portion of Area de Conservación Guanacaste (ACG) in northwestern Costa Rica (Fernandez-Triana et al. 2014, Fleming et al. 2014a, Janzen and Hallwachs 2011, Janzen et al. 2009, Rodriguez et al. 2012, Smith et al. 2012, Smith et al. 2007, Smith et al. 2006, Smith et al. 2008).The methods employed for rearing the caterpillar parasitoids are illustrated at http://janzen.bio.upenn.edu/caterpillars/methodology/how/parasitoid_husbandry.htm. In brief, caterpillars of all instars (and sometimes pupae) are found in the wild by a wide variety of search methods, and reared in captivity on the food plant species on which they were found, until they produce an adult parasitoid or die of other causes.

It should be noted that this inventory searches some vegetation types and vertical strata much more thoroughly than others. This bias is due to the methods employed for collecting of specimens, which rely solely on those animals within reach of the collectors, up to 3m above the ground. Recent comparisons of reared species of parasitoids with those collected by net or Malaise traps demonstrate that, to date, the estimated 1100+ species of tachinid flies reared by the caterpillar inventory represent less than half the species of caterpillar-parasitizing Tachinidae present in ACG. The largest unsampled habitat is the foliage of the canopy at approximately 3–4m above the ground.

This paper on *Itaplectops* is part of a larger effort to describe the new species reared during the ACG inventory (Fleming et al. 2014a, Fleming et al. 2014b, Fleming et al. 2015). This series of papers will represent the baseline for later, more detailed ecological and behavioral accounts and studies that will extend across ACG ecological groups, whole ecosystems, and taxonomic assemblages much larger than a genus.

### Dissections and imaging

Habitus and terminalia photographs were taken using the methods outlined in Fleming et al. (2014a). Raw image files were first processed with Adobe Photoshop CS6, then digitally stacked to produce a final composite image using Zerene Stacker Software v1.04. Whenever possible, the images provided are of the holotype; however, in cases where a paratype was photographed this is mentioned in the image caption.

Adult flies were dissected following standard practice (O’Hara 1983). Preparations were mounted on a depression slide in a small quantity of Rexall hand sanitizer gel (NPN# 80007138) (Fleming et al. 2014a). After mounting and photographing, the terminalia were rinsed in a small quantity of pure distilled water before being replaced in a glycerine-filled microvial.

The terminology used for structures of the terminalia (which refers here only to the sclerotized parts of the genitalia, and not to the soft internal structures) and other body parts follows Cumming and Wood (2009).The frontal vitta and fronto-orbital plate were measured at the widest point of the frontal vitta, directly above the lunule.

### Voucher specimen management

All caterpillars reared from the ACG efforts receive a unique voucher code in the format yy–SRNP–xxxxx. Any parasitoid emerging from a caterpillar receives the same voucher code. When the parasitoid is later dealt with individually, it receives a second voucher code unique to it, in the format DHJPARxxxxxxx. The voucher codes and data assigned to both host and emerged parasitoids are available at http://janzen.bio.upenn.edu/caterpillars/database.lasso. To date, all DHJPARxxxxxxx-coded tachinids have had one leg removed for attempted DNA barcoding at the Biodiversity Institute of Ontario (BIO), University of Guelph, with all collateral data and all successful barcodes permanently and publicly deposited in the Barcode of Life Data System (BOLD, www.boldsystems.org) (Ratnasingham and Hebert 2007), and later migrated to GenBank as well.

Inventoried Tachinidae were collected under Costa Rican government research permits granted to DHJ since 1978, and likewise exported under permit by DHJ from Costa Rica to Philadelphia, and from there to their final depository in the CNC. Tachinid identifications for the inventory were coordinated by DHJ with help from: AJF and DMW (morphology), MAS (barcoding), and DHJ and WH (correlation with host species). Dates of capture of each reared flies in the inventory are the dates of eclosion of the fly, not the date of capture of the caterpillar. The fly eclosion date is much more representative of the time when that fly species is on the wing, than is the date of capture of the caterpillar or, (rarely) the finding of the parasitized pupa. The collector listed is the parataxonomist who found the caterpillar rather than the person who retrieved the newly eclosed fly from its rearing bag or bottle. Life history, biology and parasitization frequencies by these flies will be the detailed subject of later papers.

The five patronyms proposed here are in honor of the Palola-McSweeney family of Vermont, USA, in recognition of their essential role in the development of the Guanacaste Dry Forest Conservation Fund (http://www.gdfcf.org) in support of Area de Conservación Guanacaste (http://www.acguanacaste.ac.cr).

The holotypes of the new species have been deposited in CNC.

### DNA barcoding

DNA barcodes (the standard 5’ region of the mitochondrial cytochrome *c* oxidase I (COI) gene) for all ACG inventory specimens were obtained using DNA extractions made from single legs using a glass fiber protocol (Ivanova et al. 2006). Total genomic DNA was re-suspended in 30μl of dH_2_O, and a 658-bp region near the 5’ terminus of the COI gene was amplified using standard primers (LepF1–LepR1), following established protocols (Smith et al. 2006, Smith et al. 2007, Smith et al. 2008). All information for the sequences associated with each individual specimen (including GenBank and BOLD accession numbers) can be retrieved from the Barcode of Life Data System (BOLD) (Ratnasingham and Hebert 2007) via the publicly available dataset dx.doi.org/10.5883/DS-ASITAPLE. A neighbor–joining (NJ) tree (Saitou and Nei 1987) for all *Itaplectops* reared and DNA-barcoded within the inventory through 2015 is included as Supplementary material in this paper (Suppl. material 1).

### Species previously included in *Itaplectops*

*Itaplectops* Townsend 1927: 265. Type species: *Itaplectops antennalis* Townsend 1927, by original designation.

*Itaplectops antennalis* Townsend 1927: 321; holotype female (USNM) [examined by DMW]. Type locality: Brazil, São Paulo, São Paulo, Cantareira. Type label: “Tremembe/23.X.//Type//*Itaplectops*/ *antennalis* /TT. ♀/ Det CHTT”.

Note: Townsend's original locality label displays the place name: "Tremembe" (sic.), however, the original publication states the type locality as Cantareira. Townsend created some confusion with the inconsistent use of the type locality 'Tremembé da Cantareira' referring to what was at his time was a railway station on the northern edge of São Paulo. Among the names he used to refer to this locality were: 'Cantareira', 'Tremembé', and 'S. Cantareira'. Tremembé da Cantareira is situated in what is now called Bairro do Tremembé, on the northern edge of the district of Tremembé. Serra do Cantareira is now used to refer to the state park that surrounds the area of the old Tremembé railway station and is likely to be the area to which Townsend was referring.

### Diagnosis of genus *Itaplectops*

Males and females ranging in total body length from 4 to 7mm.

**Head**: as wide as thorax when viewed dorsally; facial margin not visible in profile; tawny to brown frontal vitta; eyes densely haired; ocellar bristles small or absent; palpus and proboscis orange; antenna ranging from orange to dark brown, arista bare and ranging in color from orange to entirely dark brown; fronto-orbital plate silvery to very slightly gold tinged; both males and females possessing proclinate orbital bristles (these on rare occasions absent or reduced in males); parafacial and gena silvery to slightly brassy in color; parafacial bare to sparsely haired; facial ridge ranging from bare to bristled along half of its length; gena 1/4 height of eye; vibrissa strongly developed, level or almost level with facial margin. **Thorax**: thorax and scutellum light gray tomentose throughout; 4 thoracic vittae almost invisible, only slightly darker than the gray thorax, in most cases only visible under certain angles of light; prescutum 1/2–3/4 length of scutum; 3 postsutural dorsocentral bristles; 2–3 postsutural supra-alar bristles; when 3 are present then first postsutural supra-alar greatly reduced; scutellum with at least two pairs of divergent subapical bristles and one pair of crossed apicals; scutellum with 0 to 2 pairs of discal bristles; 2 katepisternal bristles, anteriormost reduced in size; legs varying from yellowish to entirely black and densely haired; wings ranging from smoky yellow to smoky gray, slightly darker along costal margin; costal spine absent; wing veins bare except for the presence of 2–5 small setulae at the base of R_4+5_; calypters translucent to clear with dense short hairs along their margins. **Abdomen:** shiny black with a varying degree of silver tomentosity along upper segmental margins; in most cases the abdominal tergites lack median discal bristles, however, when present, they only occur up to T3; mid-dorsal depression extending 1/2–2/3 distance to margin of T1+2. **Male terminalia:** cercus digitiform, elongate and minorly clubbed at apex, apical 1/3 tapering rapidly down to 1/5 at its widest point, convex when viewed laterally, not fused medially, apex of cerci ranging from very slightly divided to touching when viewed dorsally; surstylus from 7/10 to 9/10 the length of the cercus, from slightly inwardly bent apically to straight, bearing a slight hook at tip, visible in profile, surstylus ranging from bare to densely bristled. Epandrium hirsute, with two apically crossed bristles. Sternite 5 uniform across all species, lobe of sternite 5 with rough serrated inner edge, lobe covered in dense stout bristles. L-shaped hinged phallus, with the end of the basiphallus overlapping the base of the distiphallus.

### Discussion of tribal placement

Guimarães (1971) placed the genus *Itaplectops* in the tribe Blondeliini in the subfamily Exoristinae. Current evidence indicates that *Itaplectops* belongs in the tribe Uramyini of the subfamily Dexiinae, which to date included only *Thelairaporia* and *Uramya*. The Dexiinae are particularly easy to recognize due to the presence of a shared common character, namely a hinge in the phallus, accompanied with a close association of the postgonites with the base of the phallus (Wood and Zumbado 2010). Guimarães (1980) provided a diagnosis of the tribe Uramyini. He described it as a New World tribe, particularly well represented in the Neotropics, parasitizing caterpillars within the families Arctiidae (now Arctiinae in the Erebidae), Limacodidae, Megalopygidae, Lasiocampidae, and Dalceridae (Arnaud 1978, Guimarães 1980, Wood and Zumbado 2010). Guimarães (1980) provided a strong definition of the tribe based on the following diagnostic characters: general body plan slightly to strongly elongate, with body mainly black to dark brown; eyes densely haired; facial margin flat, not visible in profile; lowermost frontal bristles not extending below level of pedicel; facial ridge ranging from bare to bristled on lower half; first flagellomere elongated, nearly reaching lower facial margin; vibrissa strongly developed, almost level with lower facial margin; gena approximately 1/4 of eye height; genal dilation not well developed; prosternum and propleura devoid of strong bristles; postpronotum with four to five strong bristles; prescutum almost as long as scutum (between 2/3 and 3/4 length of scutum in *Itaplectops*); 3 postsutural dorsocentrals; 3 postsutural supra-alars (anteriormost pair weak or absent in some *Itaplectops*); wings elongate with a very faint tinge around the costa (in some species of *Uramya* the wings can be adorned with elaborate markings); costal spine absent; all tergites bearing median marginal bristles, abdominal discal bristles present, on T3 to T5, and sometimes on T1+2 (except in *Itaplectops*, which can have more varied abdominal chaetotaxy, some species lacking median marginals on T1+2 and T3, and ranging from having no discal bristles to discal bristles being present only on T1+2 and T3). Male genitalia with an L-shaped phallus, end of basiphallus prolonged over the base of the distiphallus, and a long anteriorly curved postgonite. Other similarities shared by members of the tribe, not mentioned by Guimarães (1980), include: the similarity in the arrangement of the lappets of the posterior spiracle – in all three genera the lappets of the posterior thoracic spiracle are almost equal in size, giving the spiracular opening an almost "V" shape – and a bare prosternum; in *Uramya* the prosternum appears pollinose yet devoid of bristles.

The male terminalia, which are sometimes useful in defining the subfamilies or tribes of the Tachinidae, appear as a major defining character of the tribe Uramyini. In the male terminalia, *Itaplectops* shares the synapomorphy of the L-shaped hinged phallus, with the end of the basiphallus overlapping the base of the distiphallus, traits common to the rest of the tribe. These structures are stereotypical of the terminalia in the subfamily Dexiinae and are consistent with Guimarães’s diagnosis of the male terminalia in the Uramyini (*Uramya* and *Thelairaporia)*.

Chaetotaxy, while extremely useful, is not the only important character set defining a genus (Wood 1985). *Itaplectops* differs somewhat from Guimarães's concept of Uramyini by the presence of 2 rather than 3 katepisternals, and the reduction to sometimes apparent absence of the anterior postsutural supra-alar bristle. Our results suggest a close relationship between *Itaplectops* and *Thelairaporia*, which was hinted at by Wood and Zumbado (2010). *Itaplectops* can be differentiated from *Thelairaporia* by the presence of only 2 katepisternal bristles, the presence of discals on the scutellum, and the absence of discals on T4. Genera are commonly thought of as neatly categorized assemblages of species. This idea, however, fails to acknowledge the fact that evolution is a fluid matrix in which species are constantly being created and destroyed. The proximity of these two genera may one day be clarified as existing somewhere along this sliding scale idea of evolution.

### Barcoding Results

**Figure 1.**
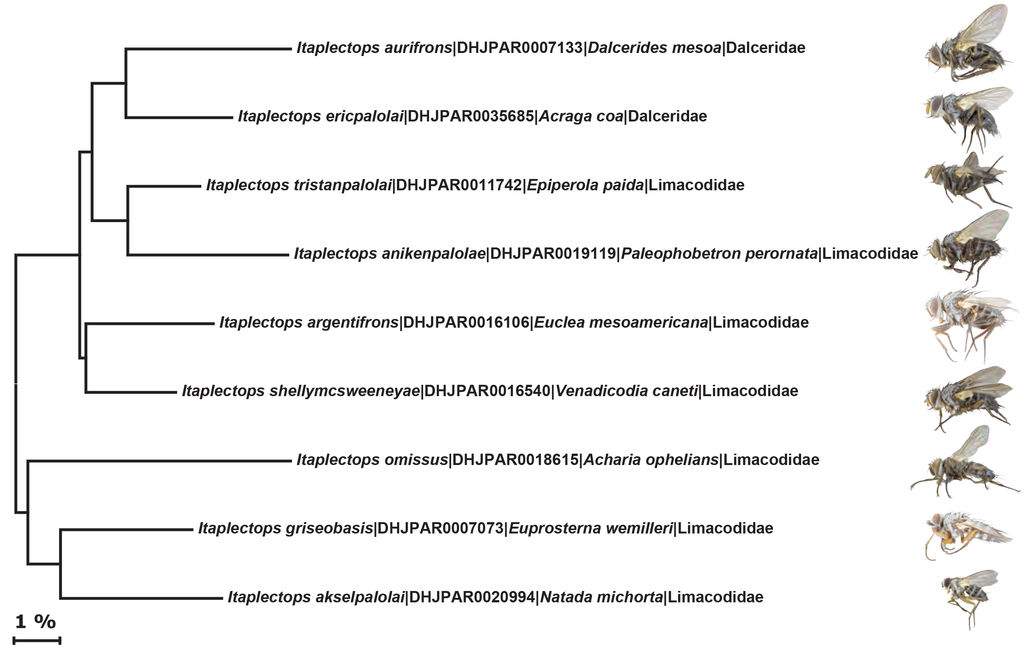
Neighbor-Joining (NJ – Saitou and Nei 1987) tree based on Kimura 2-parameter distances (K2P – Kimura 1980) made using MEGA6 (Tamura et al. 2013) for a single specimen from each of the nine species of *Itaplectops* in ACG. Tip labels include species name, specimen accession number, host species, host family and the lateral image of a male.

The DNA barcode sequences recovered from ACG *Itaplectops* display the characteristic strong AT bias of insect mitochondrial DNA (mean percent GC content 30.82, SE 0.17) and no insertions or deletions. Within-species variation was low (mean distance of 0.27%) compared to between-species variation (mean distance 8.06%). All values of DNA barcode variation were calculated within BOLD and can be re-calculated in the future as more specimens are recovered from the ACG inventory and added to the DNA library. Fig. 1 is a neighbor–joining tree for the *Itaplectops* holotypes reared and DNA-barcoded by this inventory to date.

## Taxon treatments

### Itaplectops akselpalolai

Fleming & Wood, 2014 sp. n.

urn:lsid:zoobank.org:act:067C8099-35A3-42FA-A97C-A4527532CF5B

#### Materials

- **Type status:** Holotype. **Occurrence:** occurrenceDetails: http://janzen.sas.upenn.edu; catalogNumber: DHJPAR0053409; recordedBy: D.H. Janzen & W. Hallwachs, Keiner Aragon; individualID: DHJPAR0053409; individualCount: 1; sex: M; lifeStage: adult; preparations: pinned; otherCatalogNumbers: ASHYM2763-13, 13-SRNP-79398; **Taxon:** scientificName: Itaplectops akselpalolai; phylum: Arthropoda; class: Insecta; order: Diptera; family: Tachinidae; genus: Itaplectops; specificEpithet: akselpalolai; scientificNameAuthorship: Fleming & Wood; **Location:** continent: Central America; country: Costa Rica; countryCode: CR; stateProvince: Guanacaste; county: Area de Conservacion Guanacaste; locality: Sector Rincon Rain Forest; verbatimLocality: Palomo; verbatimElevation: 96; verbatimLatitude: 10.96187; verbatimLongitude: -85.28045; verbatimCoordinateSystem: Decimal; decimalLatitude: 10.96187; decimalLongitude: -85.28045; **Identification:** identifiedBy: AJ Fleming; dateIdentified: 2014; **Event:** samplingProtocol: reared from caterpillar of *Natada michorta* (Limacodidae); verbatimEventDate: 26-Sep-2013; **Record Level:** language: en; institutionCode: CNC; collectionCode: Insects; basisOfRecord: Pinned Specimen

#### Description

**Male**

**Length:** 6mm.

**Head** (Fig. 2c): proclinate orbital bristles absent; first flagellomere entirely dark, brown to brownish orange over at least 1/2 of its surface; arista dark brown over 1/2 of its length, bright orange basally, with gradual taper; first flagellomere slightly shorter than facial margin by less than half the length of the pedicel; ocellar bristles well developed, at least the length of the pedicel, and arising behind anterior ocellus; ocellar triangle bare; frontal vitta approximately 4x as wide as fronto-orbital plate; facial ridge bearing at least 3 stout supravibrissal bristles; fronto-orbital plate slightly silver; parafacial bare and entirely silver; fronto-orbital plate bare.

**Thorax** (Fig. 2a, b): three postsutural supra-alar bristles; katepisternum with 2 bristles, anteriormost reduced in size and arising directly below to slightly anterior to suture; apical scutellar bristles short, up to 1/2 the length of subapical scutellars; subapical scutellar bristles strongly divergent; scutellum with no apparent discal bristles.

**Wings** (Fig. 2a): smoky yellow.

**Legs** (Fig. 2b): ground color of at least 1/2 of femur yellow, tibia yellow, and tarsi yellow (although these may appear dark due to hirsuteness); dorso-ventral margin of hind tarsi with yellow tufts of bristles apically.

**Abdomen** (Fig. 2a, b): T1+2 with mid-dorsal depression extending 2/3 along its length, but not reaching tergal margin; silver tomentosity on margins of abdominal segments T3 and T4 only visible under certain angles of light, with these bands not extending beyond 1/3 of tergal surface; median marginal bristles present on T1+2, T3, T4 and T5; reduced discal bristles present on T3, these arising within the silver tomentose band along the tergal margin, sometimes appearing like thickened abdominal hairs.

**Terminalia** (Fig. 2d, e): both cerci tightly juxtaposed when viewed dorsally. Cercus haired along basal 2/3^rds^; appearing convex when viewed laterally with a very slight thickening apically. Surstylus 9/10 as long as cercus, when viewed dorsally outwardly convex at its center, appearing bowed with a very slight acute downward bend apically, with a very slight hook at its tip. Cercus densely bristled along its entire length. Phallus 5x as long as cercus, straight and downwardly curved.

**Female:** unknown at this time.

#### Diagnosis

*Itaplectops akselpalolai* can be distinguished by the following combination of traits: proclinate orbital bristles absent in males; first flagellomere dark brown/black over 1/2 its surface; 3 postsutural supra-alar bristles; median marginal bristles present on T1+2, T3, T4 and T5; discal bristles present at least on T3; silver tomentosity on margins of abdominal segments T1+2, T3, and T4, confined to anterior 1/3 of tergite. It can be distinguished from its most similar congener, *Itaplectops griseobasis*, following couplet 9 in the key to *Itaplectops* (below).

#### Etymology

*Itaplectops akselpalolai* is named in honor of Aksel Palola of Vermont, USA, a supporter of Eric Palola, Shelly McSweeney, Aniken Palola and Tristan Palola, and therefore of GDFCF and ACG.

#### Distribution

Costa Rica, ACG, Prov. Alajuela and Guanacaste, rain forest.

#### Ecology

##### Hosts

Reared from caterpillars of Limacodidae, *Natada* Walker spp.

### Itaplectops anikenpalolae

Fleming & Wood, 2014 sp. n.

urn:lsid:zoobank.org:act:E9C71B34-3012-4121-9A30-6894B36C5EB9

#### Materials

- **Type status:** Holotype. **Occurrence:** occurrenceDetails: http://janzen.sas.upenn.edu; catalogNumber: DHJPAR0019119; recordedBy: D.H. Janzen & W. Hallwachs, Gusaneros; individualID: DHJPAR0019119; individualCount: 1; sex: M; lifeStage: adult; preparations: pinned; otherCatalogNumbers: ASTAI1766-07, 94-SRNP-4269; **Taxon:** scientificName: Itaplectops anikenpalolae; phylum: Arthropoda; class: Insecta; order: Diptera; family: Tachinidae; genus: Itaplectops; specificEpithet: anikenpalolae; scientificNameAuthorship: Fleming & Wood; **Location:** continent: Central America; country: Costa Rica; countryCode: CR; stateProvince: Guanacaste; county: Area de Conservacion Guanacaste; locality: Sector Santa Rosa; verbatimLocality: Area Administrativa; verbatimElevation: 295; verbatimLatitude: 10.838; verbatimLongitude: -85.619; verbatimCoordinateSystem: Decimal; decimalLatitude: 10.838; decimalLongitude: -85.619; **Identification:** identifiedBy: AJ Fleming; dateIdentified: 2014; **Event:** samplingProtocol: reared from caterpillar of *Paleophobetron perornata* (Limacodidae); verbatimEventDate: Jun-28-1994; **Record Level:** language: en; institutionCode: CNC; collectionCode: Insects; basisOfRecord: Pinned Specimen

#### Description

**Male**

**Length:** 5.5 mm.

**Head** (Fig. 3c): proclinate orbital bristles present in male; first flagellomere entirely dark brownish orange over at least 1/2 of its surface; arista dark brown over 2/3 of its length; first flagellomere slightly shorter than facial margin by a distance not exceeding the length of the pedicel; ocellar bristles reduced, almost hair-like, no longer than length of pedicel, arising behind anterior ocellus; ocellar triangle covered in small proclinate hairs; frontal vitta approximately 2x as wide as fronto-orbital plate; frontal vitta covered in fine hairs; facial ridge bearing 5–6 stout decumbent bristles; fronto-orbital plate and parafacial entirely silver with row of fine bristles; fronto-orbital plate of male with fine hairs over its entire surface, interspersed throughout and lateral to frontal bristles, these not extending past upper margin of pedicel.

**Thorax** (Fig. 3a): two postsutural supra-alar bristles; katepisternum with 2 bristles, anteriormost reduced in size and arising slightly behind suture; apical scutellar bristles long, up to 3/4 length of subapical scutellars; subapical scutellar bristles parallel or convergent (often crossed); scutellum with 1 or 2 pairs of widely separated discal bristles.

**Wings** (Fig. 3a): smoky yellow.

**Legs** (Fig. 3b): legs appearing dark overall, ground color of 1/2 of femur yellow, tibia yellow, and tarsi dark; dorso-ventral margin of hind tarsi with yellow tufts of bristles apically.

**Abdomen** (Fig. 3a, b): T1+2 with mid-dorsal depression extending along 2/3 of its length, but not reaching tergal margin; median marginal bristles present on T4 and T5, but absent on T1+2 and T3; discal bristles absent; silver tomentosity on margins of abdominal segments T3, T4 and T5 only visible under certain angles, and not extending beyond 1/3 of tergal surface.

**Terminalia** (Fig. 3d, e): cerci in posterior view tightly juxtaposed basally but slightly diverging apically, haired up to tapering point, after tapering point becoming almost uniformly wide and bare until the tip; straight when viewed laterally; surstylus 5/8 the length of the cercus, in lateral view cercus, appears curved apically giving it a hook at its tip; cercus lightly bristled along its entire length; phallus complex, 2x as long as cercus, with a downward bend.

**Female:** unknown at this time.

#### Diagnosis

*Itaplectops anikenpalolae* can be distinguished by the following combination of traits: proclinate orbital bristles present in male; first flagellomere brown/black over at least 1/2 of surface; fronto-orbital plate with small hairs interspersed throughout; 2 postsutural supra-alar bristles; median marginal bristles absent on T1+2 and T3, present on T4 and T5; discal bristles absent from all abdominal tergites; silver tomentosity present along margin of abdominal segments T3, T4, and T5. It can be distinguished from its most similar congener, *Itaplectops tristanpalolai*, following couplet 5 in the key to *Itaplectops* (below).

#### Etymology

*Itaplectops anikenpalolae* is named in honor of Aniken Palola of Vermont, USA, a supporter of Eric Palola and Shelly McSweeney, and therefore of GDFCF and ACG.

#### Distribution

Costa Rica, ACG, Prov. Guanacaste, dry forest.

#### Ecology

##### Hosts

Reared from caterpillar of the Limacodidae, *Paleophobetron perornata* (Dyar, 1905).

### Itaplectops antennalis

Townsend, 1927

http://n2t.net/ark:/65665/3ad88fd7b-9883-4620-b492-bfeaabf23e29

#### Materials

- **Type status:** Holotype. **Occurrence:** individualCount: 1; sex: F; lifeStage: Adult; **Location:** countryCode: BR; stateProvince: São Paulo; county: Brazil; municipality: Cantareira; locality: Cantareira, S. P., Brazil; **Identification:** identifiedBy: C.H.T. Townsend; **Event:** verbatimEventDate: 23 de Out.; **Record Level:** institutionCode: USNM
- **Type status:** Other material. **Occurrence:** individualCount: 1; sex: M; lifeStage: adult; **Taxon:** scientificName: Itaplectops antennalis Townsend, 1927; family: Tachinidae; genus: Itaplectops; specificEpithet: antennalis; **Location:** continent: North America; country: Mexico; countryCode: MX; stateProvince: Veracruz; locality: Jalapa; verbatimLocality: Jalapa, VC MEX; **Event:** eventDate: 1961-08-03; **Record Level:** institutionID: CNC; institutionCode: CNC
- **Type status:** Other material. **Occurrence:** individualCount: 1; sex: F; lifeStage: adult; **Taxon:** scientificName: Itaplectops antennalis Townsend, 1927; family: Tachinidae; genus: Itaplectops; specificEpithet: antennalis; **Location:** continent: North America; country: Mexico; countryCode: MX; stateProvince: Veracruz; locality: Jalapa; verbatimLocality: Jalapa, VC MEX; **Event:** eventDate: 1961-08-03; **Record Level:** institutionID: CNC; institutionCode: CNC
- **Type status:** Other material. **Occurrence:** individualCount: 1; sex: M; lifeStage: adult; **Taxon:** scientificName: Itaplectops antennalis Townsend, 1927; family: Tachinidae; genus: Itaplectops; specificEpithet: antennalis; **Location:** continent: North America; country: Mexico; countryCode: MX; stateProvince: Veracruz; locality: Cordoba; verbatimLocality: Cordoba Mex Vera Cruz; **Event:** eventDate: 1966-07-13; **Record Level:** institutionID: CNC; institutionCode: CNC
- **Type status:** Other material. **Occurrence:** individualCount: 1; sex: M; lifeStage: adult; disposition: abdomen and genitalia dissected out and glued to locality label; **Taxon:** scientificName: Itaplectops antennalis Townsend, 1927; family: Tachinidae; genus: Itaplectops; specificEpithet: antennalis; **Location:** continent: North America; country: Mexico; countryCode: MX; stateProvince: Veracruz; locality: Cordoba; verbatimLocality: Cordoba Mex Vera Cruz; **Event:** eventDate: 1966-07-13; **Record Level:** institutionID: CNC; institutionCode: CNC
- **Type status:** Other material. **Occurrence:** individualCount: 1; sex: F; lifeStage: adult; **Taxon:** scientificName: Itaplectops antennalis Townsend, 1927; family: Tachinidae; genus: Itaplectops; specificEpithet: antennalis; **Location:** continent: North America; country: Mexico; countryCode: MX; stateProvince: Veracruz; locality: Cordoba; verbatimLocality: Cordoba Mex Vera Cruz; **Event:** eventDate: 1966-07-13; **Record Level:** institutionID: CNC; institutionCode: CNC
- **Type status:** Other material. **Occurrence:** individualCount: 1; sex: F; lifeStage: adult; **Taxon:** scientificName: Itaplectops antennalis Townsend, 1927; family: Tachinidae; genus: Itaplectops; specificEpithet: antennalis; **Location:** continent: North America; country: Mexico; countryCode: MX; stateProvince: Veracruz; locality: Cordoba; verbatimLocality: Cordoba Mex Vera Cruz; **Event:** eventDate: 1966-07-13; **Record Level:** institutionID: CNC; institutionCode: CNC

#### Description

**Male and female**

**Length:** 5–6mm.

**Head** (Fig. 4c): proclinate orbital bristles present in both males and females, 3 stong pairs in male; first flagellomere brilliant pale orange; arista brilliant pale orange at its base and darkening to brown at its tip, with gradual taper; first flagellomere slightly shorter than facial margin by a distance not exceeding the length of the pedicel; ocellar bristles proclinate and reduced, but not so much as to be considered hair-like, 1.5x length of pedicel, arising behind anterior ocellus; ocellar triangle bearing few short hairs; frontal vitta 2x as wide as fronto-orbital plate; facial ridge bearing 4–8 stout decumbent bristles; fronto-orbital plate and parafacial brassy gray; parafacial bare; fronto-orbital plate of both sexes haired; male with two rows of fine hairs intermingled with, and lateral to, frontal bristles, these not extending past upper margin of pedicel.

**Thorax** (Fig. 4a, c): three postsutural supra-alar bristles, anteriormost greatly reduced; katepisternum with 2 bristles, anteriormost arising directly below suture; apical scutellar bristles long, 1/2–3/4 the length of subapical scutellars; subapical scutellar bristles parallel or convergent (often crossed); scutellum with 1–2 pairs of widely separated discal bristles.

**Wings** (Fig. 4a, b): smoky brown.

**Legs** (Fig. 4b): appearing dark overall, femur yellow at joint with tibia, tibia and tarsi yellow.

**Abdomen**(Fig. 4a, b): T1+2 with mid-dorsal depression extending halfway along its length, not reaching tergal margin; median marginal bristles present on T4 and T5, but absent on T1+2 and T3. Discal bristles absent. Silver tomentosity on margins of abdominal segments T3 and T4 not extending beyond 1/3 of tergal surface.

**Male terminalia** (not pictured, examined *in situ*): cerci tightly juxtaposed when viewed dorsally; haired up to tapering point, then bare until the tip; apparently convex when viewed laterally; apically clubbed; surstylus 4/5 the length of the cercus, outwardly convex at its center so as to appear outwardly bowed with an apically hooked tip, visible in lateral view; densely bristled along its entire length; phallus 1.5x as long as cercus, with a downward bend.

#### Diagnosis

*Itaplectops antennalis* can be distinguished by the following combination of traits: proclinate orbital bristles present in males; first flagellomere brilliant pale orange; parafacial bare; median marginal bristles absent on T1+2 and T3 but present on T4 and T5; discal bristles absent from all tergites; silver tomentosity present on margins of abdominal segments T3 and T4. It can be distinguished from its most similar congener, *Itaplectops ericpalolai*, following couplet 3 in the key to *Itaplectops* (below).

#### Distribution

Brazil: São Paulo, Cantareira; Mexico: Veracruz, Cordoba, Jalapa.

### Itaplectops argentifrons

Fleming & Wood, 2014 sp. n.

urn:lsid:zoobank.org:act:B8B22BAF-46B8-4544-B7E5-9061A72A1445

#### Materials

- **Type status:** Holotype. **Occurrence:** occurrenceDetails: http://janzen.sas.upenn.edu; catalogNumber: 11-SRNP-32160; recordedBy: D.H. Janzen & W. Hallwachs, Minor Carmona; individualID: 11-SRNP-32160; individualCount: 1; sex: M; lifeStage: adult; preparations: pinned; **Taxon:** scientificName: Itaplectops argentifrons; phylum: Arthropoda; class: Insecta; order: Diptera; family: Tachinidae; genus: Itaplectops; specificEpithet: argentifrons; scientificNameAuthorship: Fleming & Wood; **Location:** continent: Central America; country: Costa Rica; countryCode: CR; stateProvince: Alajuela; county: Area de Conservacion Guanacaste; locality: Sector Rincon Rain Forest; verbatimLocality: Puente Rio Negro; verbatimElevation: 675; verbatimLatitude: 10.989; verbatimLongitude: -85.426; verbatimCoordinateSystem: Decimal; decimalLatitude: 10.989; decimalLongitude: -85.426; **Identification:** identifiedBy: AJ Fleming; dateIdentified: 2014; **Event:** samplingProtocol: reared from caterpillar of *Euclea mesoamericana* (Limacodidae); verbatimEventDate: 25-Aug-2011; **Record Level:** language: en; institutionCode: CNC; collectionCode: Insects; basisOfRecord: Pinned Specimen
- **Type status:** Paratype. **Occurrence:** occurrenceDetails: http://janzen.sas.upenn.edu; catalogNumber: 06-SRNP-43634; recordedBy: D.H. Janzen & W. Hallwachs, Freddy Quesada; individualID: 06-SRNP-43634; individualCount: 1; sex: M; lifeStage: adult; preparations: pinned; **Taxon:** scientificName: Itaplectops argentifrons; phylum: Arthropoda; class: Insecta; order: Diptera; family: Tachinidae; genus: Itaplectops; specificEpithet: argentifrons; scientificNameAuthorship: Fleming & Wood; **Location:** continent: Central America; country: Costa Rica; countryCode: CR; stateProvince: Guanacaste; county: Area de Conservacion Guanacaste; locality: Sector Pitilla; verbatimLocality: Estación Pitilla; verbatimElevation: 340; verbatimLatitude: 10.904; verbatimLongitude: -85.303; verbatimCoordinateSystem: Decimal; decimalLatitude: 10.904; decimalLongitude: -85.303; **Identification:** identifiedBy: AJ Fleming; dateIdentified: 2014; **Event:** samplingProtocol: reared from caterpillar of *Euclea mesoamericana* (Limacodidae); verbatimEventDate: 09-Oct-2006; **Record Level:** language: en; institutionCode: CNC; collectionCode: Insects; basisOfRecord: Pinned Specimen
- **Type status:** Paratype. **Occurrence:** occurrenceDetails: http://janzen.sas.upenn.edu; catalogNumber: DHJPAR0016106; recordedBy: D.H. Janzen & W. Hallwachs, Freddy Quesada; individualID: DHJPAR0016106; individualCount: 1; sex: M; lifeStage: adult; preparations: pinned; otherCatalogNumbers: ASTAP135-06, 06-SRNP-43634; **Taxon:** scientificName: Itaplectops argentifrons; phylum: Arthropoda; class: Insecta; order: Diptera; family: Tachinidae; genus: Itaplectops; specificEpithet: argentifrons; scientificNameAuthorship: Fleming & Wood; **Location:** continent: Central America; country: Costa Rica; countryCode: CR; stateProvince: Guanacaste; county: Area de Conservacion Guanacaste; locality: Sector Pitilla; verbatimLocality: Estación Pitilla; verbatimElevation: 340; verbatimLatitude: 10.904; verbatimLongitude: -85.303; verbatimCoordinateSystem: Decimal; decimalLatitude: 10.904; decimalLongitude: -85.303; **Identification:** identifiedBy: AJ Fleming; dateIdentified: 2014; **Event:** samplingProtocol: reared from caterpillar of *Euclea mesoamericana* (Limacodidae); verbatimEventDate: 09-Oct-2006; **Record Level:** language: en; institutionCode: CNC; collectionCode: Insects; basisOfRecord: Pinned Specimen
- **Type status:** Paratype. **Occurrence:** occurrenceDetails: http://janzen.sas.upenn.edu; catalogNumber: DHJPAR0044962; recordedBy: D.H. Janzen & W. Hallwachs, Minor Carmona; individualID: DHJPAR0044962; individualCount: 1; sex: F; lifeStage: adult; preparations: pinned; otherCatalogNumbers: ACGAZ186-11, 11-SRNP-32160; **Taxon:** scientificName: Itaplectops argentifrons; phylum: Arthropoda; class: Insecta; order: Diptera; family: Tachinidae; genus: Itaplectops; specificEpithet: argentifrons; scientificNameAuthorship: Fleming & Wood; **Location:** continent: Central America; country: Costa Rica; countryCode: CR; stateProvince: Alajuela; county: Area de Conservacion Guanacaste; locality: Sector Rincon Rain Forest; verbatimLocality: Puente Rio Negro; verbatimElevation: 675; verbatimLatitude: 10.989; verbatimLongitude: -85.426; verbatimCoordinateSystem: Decimal; decimalLatitude: 10.989; decimalLongitude: -85.426; **Identification:** identifiedBy: AJ Fleming; dateIdentified: 2014; **Event:** samplingProtocol: reared from caterpillar of *Euclea mesoamericana* (Limacodidae); verbatimEventDate: 25-Aug-2011; **Record Level:** language: en; institutionCode: CNC; collectionCode: Insects; basisOfRecord: Pinned Specimen

#### Description

**Males and females**

**Length:** male 4–5mm; female 5mm.

**Head** (Fig. 5c): proclinate orbital bristles present in both males and females; first flagellomere entirely dark or brownish orange over at least 1/2 of its surface; arista dark brown over 1/2 of its length, with gradual taper; first flagellomere reaching facial margin; ocellar bristles reduced, almost hair-like, no longer than length of pedicel, arising behind anterior ocellus; ocellar triangle covered in small proclinate hairs; frontal vitta approximately 2x as wide as fronto-orbital plate; facial ridge bearing 3–4 stout, decumbent bristles; fronto-orbital plate and parafacial entirely silver; parafacial bare; fronto-orbital plate of male with fine hairs confined to a row lateral to frontal bristles, these not extending past lowest frontal bristle; absent in female.

**Thorax** (Fig. 5a): three postsutural supra-alar bristles, anteriormost greatly reduced to an almost hair-like structure; katepisternum with 2 bristles, anteriormost reduced in size and arising slightly behind suture; apical scutellar bristles long, up to 3/4 length of subapical scutellars; subapical scutellar bristles parallel or convergent (often crossed); scutellum with 1–2 pairs of widely separated discal bristles.

**Wings** (Fig. 5a): smoky yellow.

**Legs** (Fig. 5b): appearing dark overall, at least 1/2 of femur yellow, tibia yellow, and tarsi yellow (although these may appear dark due to hirsuteness); dorso-ventral margin of hind tarsi with yellow tufts of bristles apically.

**Abdomen** (Fig. 5a, b): T1+2 with mid-dorsal depression extending along 2/3 of its length, but not reaching tergal margin; median marginal bristles present on T4 and T5 but absent on T1+2 and T3; discal bristles absent from all tergites; silver tomentosity on margins of abdominal segments T3 and T4 only visible under certain angles, and not extending beyond 1/3 of tergal surface.

**Male terminalia** (Fig. 5d, e): both cerci tightly juxtaposed basally and diverging at their tips; haired up to tapering point, and bare until the tip; cercus, in lateral view, with a downward bend at 1/3 its length then curving back upwards apically,forming a very slight hook at its tip; surstylus 9/10 the length of the cercus, outwardly convex at its center so as to appear outwardly bowed with a slight inward bend apically; surstylus vaguely “S” shaped, with a downwardly hooked tip when viewed laterally; densely bristled along its entire length; phallus 2x as long as cercus, with a downward bend.

#### Diagnosis

*Itaplectops argentifrons* can be distinguished by the following combination of traits: proclinate orbital bristles present in males; fronto-orbital plate with silver sheen; first flagellomere brown/black over at least 1/2 of surface; three postsutural supra-alar bristles, anteriormost greatly reduced; median marginal bristles absent on T1+2 and T3 but present on T4 and T5; discal bristles absent from all tergites; silver tomentosity present on margins of abdominal segments T3 and T4. It can be distinguished from its most similar congener, *Itaplectops aurifrons*, following couplet 7 in the key to *Itaplectops* (below).

#### Etymology

From the Latin adjective, *argentea*, meaning “silver-bearing”, and the Latin noun *frons*, meaning "forehead" (a common term in insect anatomy), in reference to the solid silver tomentosity of the fronto-orbital plate and parafacial.

#### Distribution

Costa Rica, ACG, Prov. Alajuela and Guanacaste, rain forest and dry forest.

#### Ecology

##### Hosts

Reared from caterpillars of the Limacodidae *Euclea mesoamericana* Corrales & Epstein, 2004.

### Itaplectops aurifrons

Fleming & Wood, 2014 sp. n.

urn:lsid:zoobank.org:act:A9672474-4A54-4556-855A-DB38CEAFD3B7

#### Materials

- **Type status:** Holotype. **Occurrence:** occurrenceDetails: http://janzen.sas.upenn.edu; catalogNumber: DHJPAR0007133; recordedBy: D.H. Janzen & W. Hallwachs, Gloria Sihezar; individualID: DHJPAR0007133; individualCount: 1; sex: M; lifeStage: adult; preparations: pinned; otherCatalogNumbers: ASTAV375-06, 06-SRNP-1262; **Taxon:** scientificName: Itaplectops aurifrons; phylum: Arthropoda; class: Insecta; order: Diptera; family: Tachinidae; genus: Itaplectops; specificEpithet: aurifrons; scientificNameAuthorship: Fleming & Wood; **Location:** continent: Central America; country: Costa Rica; countryCode: CR; stateProvince: Alajuela; county: Area de Conservacion Guanacaste; locality: Sector San Cristobal; verbatimLocality: Cementerio Viejo; verbatimElevation: 570; verbatimLatitude: 10.881; verbatimLongitude: -85.389; verbatimCoordinateSystem: Decimal; decimalLatitude: 10.881; decimalLongitude: -85.389; **Identification:** identifiedBy: AJ Fleming; dateIdentified: 2014; **Event:** samplingProtocol: reared from caterpillar of Dalcerides mesoa (Dalceridae); verbatimEventDate: Mar-04-2006; **Record Level:** language: en; institutionCode: CNC; collectionCode: Insects; basisOfRecord: Pinned Specimen
- **Type status:** Paratype. **Occurrence:** occurrenceDetails: http://janzen.sas.upenn.edu; catalogNumber: DHJPAR0023648; recordedBy: D.H. Janzen & W. Hallwachs, Dunia Garcia; individualID: DHJPAR0023648; individualCount: 1; sex: F; lifeStage: adult; preparations: pinned; otherCatalogNumbers: ASTAW505-08, 07-SRNP-47422; **Taxon:** scientificName: Itaplectops aurifrons; phylum: Arthropoda; class: Insecta; order: Diptera; family: Tachinidae; genus: Itaplectops; specificEpithet: aurifrons; scientificNameAuthorship: Fleming & Wood; **Location:** continent: Central America; country: Costa Rica; countryCode: CR; stateProvince: Guanacaste; county: Area de Conservacion Guanacaste; locality: Sector Cacao; verbatimLocality: Cuesta Caimito; verbatimElevation: 640; verbatimLatitude: 10.891; verbatimLongitude: -85.472; verbatimCoordinateSystem: Decimal; decimalLatitude: 10.891; decimalLongitude: -85.472; **Identification:** identifiedBy: AJ Fleming; dateIdentified: 2014; **Event:** samplingProtocol: reared from caterpillar of Rosema attenuata (Notodontidae); verbatimEventDate: Jan-13-2008; **Record Level:** language: en; institutionCode: CNC; collectionCode: Insects; basisOfRecord: Pinned Specimen
- **Type status:** Paratype. **Occurrence:** occurrenceDetails: http://janzen.sas.upenn.edu; catalogNumber: DHJPAR0019121; recordedBy: D.H. Janzen & W. Hallwachs, Gloria Sihezar; individualID: DHJPAR0019121; sex: F; lifeStage: adult; preparations: pinned; otherCatalogNumbers: ASTAI1768-07, 98-SRNP-6258; **Taxon:** scientificName: Itaplectops aurifrons; phylum: Arthropoda; class: Insecta; order: Diptera; family: Tachinidae; genus: Itaplectops; specificEpithet: aurifrons; scientificNameAuthorship: Fleming & Wood; **Location:** continent: Central America; country: Costa Rica; countryCode: CR; stateProvince: Alajuela; county: Area de Conservacion Guanacaste; locality: Sector San Cristobal; verbatimLocality: Sendero Corredor; verbatimElevation: 620; verbatimLatitude: 10.879; verbatimLongitude: -85.39; verbatimCoordinateSystem: Decimal; decimalLatitude: 10.879; decimalLongitude: -85.39; **Identification:** identifiedBy: AJ Fleming; dateIdentified: 2014; **Event:** samplingProtocol: reared from caterpillar of Parasa sandrae (Limacodidae); verbatimEventDate: Feb-27-1998; **Record Level:** language: en; institutionCode: CNC; collectionCode: Insects; basisOfRecord: Pinned Specimen
- **Type status:** Paratype. **Occurrence:** occurrenceDetails: http://janzen.sas.upenn.edu; catalogNumber: DHJPAR0052468; recordedBy: D.H. Janzen & W. Hallwachs, Dunia Garcia; individualID: DHJPAR0052468; individualCount: 1; sex: F; lifeStage: adult; preparations: pinned; otherCatalogNumbers: ASHYM1822-13, 13-SRNP-35378; **Taxon:** scientificName: Itaplectops aurifrons; phylum: Arthropoda; class: Insecta; order: Diptera; family: Tachinidae; genus: Itaplectops; specificEpithet: aurifrons; scientificNameAuthorship: Fleming & Wood; **Location:** continent: Central America; country: Costa Rica; countryCode: CR; stateProvince: Guanacaste; county: Area de Conservacion Guanacaste; locality: Sector Cacao; verbatimLocality: Sendero Abajo; verbatimCoordinateSystem: Decimal; **Identification:** identifiedBy: AJ Fleming; dateIdentified: 2014; **Event:** samplingProtocol: reared from caterpillar of Acharia apicalis (Limacodidae); verbatimEventDate: Jul-15-2013; **Record Level:** language: en; institutionCode: CNC; collectionCode: Insects; basisOfRecord: Pinned Specimen
- **Type status:** Paratype. **Occurrence:** occurrenceDetails: http://janzen.sas.upenn.edu; catalogNumber: DHJPAR0019122; recordedBy: D.H. Janzen & W. Hallwachs, Gloria Sihezar; individualID: DHJPAR0019122; individualCount: 1; sex: F; lifeStage: adult; preparations: pinned; otherCatalogNumbers: ASTAI1769-07, 98-SRNP-6258; **Taxon:** scientificName: Itaplectops aurifrons; phylum: Arthropoda; class: Insecta; order: Diptera; family: Tachinidae; genus: Itaplectops; specificEpithet: aurifrons; scientificNameAuthorship: Fleming & Wood; **Location:** continent: Central America; country: Costa Rica; countryCode: CR; stateProvince: Alajuela; county: Area de Conservacion Guanacaste; locality: Sector San Cristobal; verbatimLocality: Sendero Corredor; verbatimElevation: 620; verbatimLatitude: 10.879; verbatimLongitude: -85.39; verbatimCoordinateSystem: Decimal; decimalLatitude: 10.879; decimalLongitude: -85.39; **Identification:** identifiedBy: AJ Fleming; dateIdentified: 2014; **Event:** samplingProtocol: reared from caterpillar of Parasa sandrae (Limacodidae); verbatimEventDate: Feb-27-1998; **Record Level:** language: en; institutionCode: CNC; collectionCode: Insects; basisOfRecord: Pinned Specimen
- **Type status:** Paratype. **Occurrence:** occurrenceDetails: http://janzen.sas.upenn.edu; catalogNumber: DHJPAR0019123; recordedBy: D.H. Janzen & W. Hallwachs, Gloria Sihezar; individualID: DHJPAR0019123; individualCount: 1; sex: F; lifeStage: adult; preparations: pinned; otherCatalogNumbers: ASTAI1770-07, 98-SRNP-6258; **Taxon:** scientificName: Itaplectops aurifrons; phylum: Arthropoda; class: Insecta; order: Diptera; family: Tachinidae; genus: Itaplectops; specificEpithet: aurifrons; scientificNameAuthorship: Fleming & Wood; **Location:** continent: Central America; country: Costa Rica; countryCode: CR; stateProvince: Alajuela; county: Area de Conservacion Guanacaste; locality: Sector San Cristobal; verbatimLocality: Sendero Corredor; verbatimElevation: 620; verbatimLatitude: 10.879; verbatimLongitude: -85.39; verbatimCoordinateSystem: Decimal; decimalLatitude: 10.879; decimalLongitude: -85.39; **Identification:** identifiedBy: AJ Fleming; dateIdentified: 2014; **Event:** samplingProtocol: reared from caterpillar of Parasa sandrae (Limacodidae); verbatimEventDate: Feb-27-1998; **Record Level:** language: en; institutionCode: CNC; collectionCode: Insects; basisOfRecord: Pinned Specimen
- **Type status:** Paratype. **Occurrence:** occurrenceDetails: http://janzen.sas.upenn.edu; catalogNumber: DHJPAR0019120; recordedBy: D.H. Janzen & W. Hallwachs, gusaneros; individualID: DHJPAR0019120; individualCount: 1; sex: M; lifeStage: adult; preparations: pinned; otherCatalogNumbers: ASTAI1767-07, 93-SRNP-2131; **Taxon:** scientificName: Itaplectops aurifrons; phylum: Arthropoda; class: Insecta; order: Diptera; family: Tachinidae; genus: Itaplectops; specificEpithet: aurifrons; scientificNameAuthorship: Fleming & Wood; **Location:** continent: Central America; country: Costa Rica; countryCode: CR; stateProvince: Guanacaste; county: Area de Conservacion Guanacaste; locality: Sector Santa Rosa; verbatimLocality: Cafetal; verbatimElevation: 280; verbatimLatitude: 10.858; verbatimLongitude: -85.611; verbatimCoordinateSystem: Decimal; decimalLatitude: 10.858; decimalLongitude: -85.611; **Identification:** identifiedBy: AJ Fleming; dateIdentified: 2014; **Event:** samplingProtocol: reared from caterpillar of Parasa wellesca (Limacodidae); verbatimEventDate: Jun-29-1993; **Record Level:** language: en; institutionCode: CNC; collectionCode: Insects; basisOfRecord: Pinned Specimen
- **Type status:** Paratype. **Occurrence:** occurrenceDetails: http://janzen.sas.upenn.edu; catalogNumber: DHJPAR0016104; recordedBy: D.H. Janzen & W. Hallwachs, Wilberth Araya Alegria; individualID: DHJPAR0016104; individualCount: 1; sex: M; lifeStage: adult; preparations: pinned; otherCatalogNumbers: ASTAP133-06, 06-SRNP-33945; **Taxon:** scientificName: Itaplectops aurifrons; phylum: Arthropoda; class: Insecta; order: Diptera; family: Tachinidae; genus: Itaplectops; specificEpithet: aurifrons; scientificNameAuthorship: Fleming & Wood; **Location:** continent: Central America; country: Costa Rica; countryCode: CR; stateProvince: Guanacaste; county: Area de Conservacion Guanacaste; locality: Sector Pitilla; verbatimLocality: Sendero Laguna; verbatimElevation: 680; verbatimLatitude: 10.989; verbatimLongitude: -85.423; verbatimCoordinateSystem: Decimal; decimalLatitude: 10.989; decimalLongitude: -85.423; **Identification:** identifiedBy: AJ Fleming; dateIdentified: 2014; **Event:** samplingProtocol: reared from caterpillar of Vipsophobetron marisa (Limacodidae); verbatimEventDate: Sep-29-2006; **Record Level:** language: en; institutionCode: CNC; collectionCode: Insects; basisOfRecord: Pinned Specimen
- **Type status:** Paratype. **Occurrence:** occurrenceDetails: http://janzen.sas.upenn.edu; catalogNumber: DHJPAR0055013; recordedBy: D.H. Janzen & W. Hallwachs, Ricardo Calero; individualID: DHJPAR0055013; individualCount: 1; sex: M; lifeStage: adult; preparations: pinned; otherCatalogNumbers: ASHYH1560-14, 14-SRNP-70421; **Taxon:** scientificName: Itaplectops aurifrons; phylum: Arthropoda; class: Insecta; order: Diptera; family: Tachinidae; genus: Itaplectops; specificEpithet: aurifrons; scientificNameAuthorship: Fleming & Wood; **Location:** continent: Central America; country: Costa Rica; countryCode: CR; stateProvince: Guanacaste; county: Area de Conservacion Guanacaste; locality: Sector Pitilla; verbatimLocality: Sendero Manguera; verbatimCoordinateSystem: Decimal; **Identification:** identifiedBy: AJ Fleming; dateIdentified: 2014; **Event:** samplingProtocol: reared from caterpillar of Euprosterna elaea (Limacodidae); verbatimEventDate: Mar-14-2014; **Record Level:** language: en; institutionCode: CNC; collectionCode: Insects; basisOfRecord: Pinned Specimen
- **Type status:** Paratype. **Occurrence:** occurrenceDetails: http://janzen.sas.upenn.edu; catalogNumber: DHJPAR0019124; recordedBy: D.H. Janzen & W. Hallwachs, Harry Ramirez; individualID: DHJPAR0019124; individualCount: 1; sex: M; lifeStage: adult; preparations: pinned; otherCatalogNumbers: ASTAI1771-07, 04-SRNP-46517; **Taxon:** scientificName: Itaplectops aurifrons; phylum: Arthropoda; class: Insecta; order: Diptera; family: Tachinidae; genus: Itaplectops; specificEpithet: aurifrons; scientificNameAuthorship: Fleming & Wood; **Location:** continent: Central America; country: Costa Rica; countryCode: CR; stateProvince: Guanacaste; county: Area de Conservacion Guanacaste; locality: Sector Cacao; verbatimLocality: Quebrada Otilio; verbatimElevation: 550; verbatimLatitude: 10.89; verbatimLongitude: -85.48; verbatimCoordinateSystem: Decimal; decimalLatitude: 10.89; decimalLongitude: -85.48; **Identification:** identifiedBy: AJ Fleming; dateIdentified: 2014; **Event:** samplingProtocol: reared from caterpillar of Acharia sarans (Limacodidae); verbatimEventDate: Jul-10-2004; **Record Level:** language: en; institutionCode: CNC; collectionCode: Insects; basisOfRecord: Pinned Specimen
- **Type status:** Paratype. **Occurrence:** occurrenceDetails: http://janzen.sas.upenn.edu; catalogNumber: DHJPAR0052467; recordedBy: D.H. Janzen & W. Hallwachs, Dunia Garcia; individualID: DHJPAR0052467; individualCount: 1; sex: M; lifeStage: adult; preparations: pinned; otherCatalogNumbers: ASHYM1821-13, 13-SRNP-35377; **Taxon:** scientificName: Itaplectops aurifrons; phylum: Arthropoda; class: Insecta; order: Diptera; family: Tachinidae; genus: Itaplectops; specificEpithet: aurifrons; scientificNameAuthorship: Fleming & Wood; **Location:** continent: Central America; country: Costa Rica; countryCode: CR; stateProvince: Guanacaste; county: Area de Conservacion Guanacaste; locality: Sector Cacao; verbatimLocality: Sendero Abajo; verbatimCoordinateSystem: Decimal; **Identification:** identifiedBy: AJ Fleming; dateIdentified: 2014; **Event:** samplingProtocol: reared from caterpillar of Acharia apicalis (Limacodidae); verbatimEventDate: Jul-15-2013; **Record Level:** language: en; institutionCode: CNC; collectionCode: Insects; basisOfRecord: Pinned Specimen
- **Type status:** Paratype. **Occurrence:** occurrenceDetails: http://janzen.sas.upenn.edu; catalogNumber: DHJPAR0023103; recordedBy: D.H. Janzen & W. Hallwachs, Manuel Rios; individualID: DHJPAR0023103; individualCount: 1; sex: M; lifeStage: adult; preparations: pinned; otherCatalogNumbers: ASTAW264-08, 07-SRNP-34168; **Taxon:** scientificName: Itaplectops aurifrons; phylum: Arthropoda; class: Insecta; order: Diptera; family: Tachinidae; genus: Itaplectops; specificEpithet: aurifrons; scientificNameAuthorship: Fleming & Wood; **Location:** continent: Central America; country: Costa Rica; countryCode: CR; stateProvince: Guanacaste; county: Area de Conservacion Guanacaste; locality: Sector Pitilla; verbatimLocality: Amonias; verbatimElevation: 390; verbatimLatitude: 11.042; verbatimLongitude: -85.403; verbatimCoordinateSystem: Decimal; decimalLatitude: 11.042; decimalLongitude: -85.403; **Identification:** identifiedBy: AJ Fleming; dateIdentified: 2014; **Event:** samplingProtocol: reared from caterpillar of Parasa minima (Limacodidae); verbatimEventDate: Jan-18-2008; **Record Level:** language: en; institutionCode: CNC; collectionCode: Insects; basisOfRecord: Pinned Specimen

#### Description

**Male and female**

**Length:** male 4–5mm; female 4–5mm.

**Head** (Fig. 6c): proclinate orbital bristles present in both males and females; first flagellomere entirely dark or brownish orange over at least 1/2 of its surface; arista dark brown over 1/2 of its length, bright orange basally, with gradual taper; first flagellomere reaching facial margin; ocellar bristles reduced, almost hair-like, as long or longer than length of pedicel, arising behind anterior ocellus; ocellar triangle covered in small proclinate hairs; frontal vitta approximately 2x as wide as fronto-orbital plate; facial ridge bearing at least 8 stout, decumbent bristles; fronto-orbital plate slightly gold tinged; parafacial entirely silver and bare; fronto-orbital plate of male with fine bristles confined to a row lateral to frontal bristles, these not extending past lowest frontal bristle; absent in females.

**Thorax** (Fig. 6a): three postsutural supra-alar bristles, anteriormost greatly reduced to an almost hair-like structure; katepisternum with 2 bristles, anteriormost reduced in size, arising slightly behind suture; apical scutellar bristles long, up to 3/4 length of subapical scutellars; subapical scutellar bristles parallel or convergent (often crossed); scutellum with 1–2 pairs of widely separated discal bristles.

**Wings** (Fig. 6a, b): smoky yellow.

**Legs** (Fig. 6b): appearing dark overall, femur at least 1/2 yellow, tibia yellow, and tarsi yellow (although these may appear dark due to hirsuteness); dorso-ventral margin of hind tarsi with yellow tufts of bristles apically.

**Abdomen** (Fig. 6a, b): T1+2 with mid-dorsal depression extending along 2/3 of its length, but not reaching tergal margin; median marginal bristles present on T4 and T5 but absent on T1+2 and T3. Reduced discal bristles present on T3, sometimes appearing like thicker abdominal hairs. Silver tomentosity on margins of abdominal segments T3 and T4 only visible under certain angles of light, this not extending beyond 1/3 of tergal surface.

**Male terminalia** (Fig. 6d, e): cerci tightly juxtaposed when viewed dorsally; haired up to tapering point, then bare until the tip; slightly convex when viewed laterally; surstylus almost as long as the cercus, outwardly convex at its center so as to appear outwardly bowed with a slight inward bend apically, giving it a slightly hooked tip; densely bristled along its entire length; phallus 2x as long as cercus, with a downward bend.

#### Diagnosis

*Itaplectops aurifrons* can be distinguished by the following combination of traits: proclinate orbital bristles present in males; fronto-orbital plate with gold sheen; first flagellomere black/brown over at least 1/2 its surface; three postsutural supra-alar bristles; median marginal bristles absent on T1+2 and T3 but present on T4 and T5; discal bristles present at least on T3 (in some cases these can appear reduced but still present); silver tomentosity present on margins of abdominal segments T3 and T4. It can be distinguished from its most similar congener, *Itaplectops argentifrons*, following couplet 7 in the key to *Itaplectops* (below).

#### Etymology

From the Latin adjective, *aurum*, meaning “gold”, and the Latin noun *frons*, meaning "forehead" (a common term in insect anatomy), in reference to the bright golden tomentosity on the fronto-orbital plate and parafacial.

#### Distribution

Costa Rica, ACG, Prov. Alajuela and Guanacaste, rain forest and dry forest.

#### Ecology

##### Hosts

Reared from caterpillars of the Limacodidae, *Vipsophobetron marisa* Druce, 1900.

### Itaplectops ericpalolai

Fleming & Wood, 2014 sp. n.

urn:lsid:zoobank.org:act:09DE9889-79E2-492C-AF44-BEE2F077AD6D

#### Materials

- **Type status:** Holotype. **Occurrence:** occurrenceDetails: http://janzen.sas.upenn.edu; catalogNumber: DHJPAR0035685; recordedBy: D.H. Janzen & W. Hallwachs, Jose Perez; individualID: DHJPAR0035685; individualCount: 1; sex: M; lifeStage: adult; preparations: pinned; otherCatalogNumbers: ASHYD1066-09, 09-SRNP-41309; **Taxon:** scientificName: Itaplectops ericpalolai; phylum: Arthropoda; class: Insecta; order: Diptera; family: Tachinidae; genus: Itaplectops; specificEpithet: ericpalolai; scientificNameAuthorship: Fleming & Wood; **Location:** continent: Central America; country: Costa Rica; countryCode: CR; stateProvince: Alajuela; county: Area de Conservacion Guanacaste; locality: Sector Rincon Rain Forest; verbatimLocality: Vochysia; verbatimElevation: 320; verbatimLatitude: 10.867; verbatimLongitude: -85.245; verbatimCoordinateSystem: Decimal; decimalLatitude: 10.867; decimalLongitude: -85.245; **Identification:** identifiedBy: AJ Fleming; dateIdentified: 2014; **Event:** samplingProtocol: reared from caterpillar of *Acraga coa* (Dalceridae); verbatimEventDate: 2/Jul/09; **Record Level:** language: en; institutionCode: CNC; collectionCode: Insects; basisOfRecord: Pinned Specimen
- **Type status:** Paratype. **Occurrence:** occurrenceDetails: http://janzen.sas.upenn.edu; catalogNumber: DHJPAR0035690; recordedBy: D.H. Janzen & W. Hallwachs, Jose Perez; individualID: DHJPAR0035690; individualCount: 1; sex: F; lifeStage: adult; preparations: pinned; otherCatalogNumbers: ASHYD1071-09, 09-SRNP-41310; **Taxon:** scientificName: Itaplectops ericpalolai; phylum: Arthropoda; class: Insecta; order: Diptera; family: Tachinidae; genus: Itaplectops; specificEpithet: ericpalolai; scientificNameAuthorship: Fleming & Wood; **Location:** continent: Central America; country: Costa Rica; countryCode: CR; stateProvince: Alajuela; county: Area de Conservacion Guanacaste; locality: Sector Rincon Rain Forest; verbatimLocality: Vochysia; verbatimElevation: 320; verbatimLatitude: 10.867; verbatimLongitude: -85.245; verbatimCoordinateSystem: Decimal; decimalLatitude: 10.867; decimalLongitude: -85.245; **Identification:** identifiedBy: AJ Fleming; dateIdentified: 2014; **Event:** samplingProtocol: reared from caterpillar of *Acraga coa* (Dalceridae); verbatimEventDate: 3/Jul/09; **Record Level:** language: en; institutionCode: CNC; collectionCode: Insects; basisOfRecord: Pinned Specimen

#### Description

**Male and female**

**Length:** male 5mm; female 5mm.

**Head** (Fig. 7c): proclinate orbital bristles present in both male and female; male with 3 pairs, middle pair reduced to 1/2 the length of outer 2 pairs; first flagellomere brilliant pale orange; arista brilliant pale orange at its base and darkening to brown at its tip, with gradual taper; first flagellomere slightly shorter than facial margin by a distance not exceeding the length of the pedicel; ocellar bristles reduced, almost hair-like, no longer than length of pedicel, arising behind anterior ocellus; ocellar triangle covered in small proclinate hairs; frontal vitta 2x as wide as fronto-orbital plate; facial ridge bearing 5–6 stout, decumbent bristles; fronto-orbital plate and parafacial entirely silver; parafacial with 1 bristle halfway between lowest frontal bristle and facial margin; female with parafacial bare; fronto-orbital plate of male with one row of fine bristles lateral to frontal bristles, these not extending past upper margin of pedicel; absent in female.

**Thorax** (Fig. 7b): three postsutural supra-alar bristles, anteriormost greatly reduced; katepisternum with 2 bristles, anteriormost arising slightly behind suture; apical scutellar bristles long, 3/4 the length of subapical scutellars; subapical scutellar bristles parallel or convergent (often crossed); scutellum with 1–2 pairs of widely separated discal bristles.

**Wings** (Fig. 7a): smoky yellow.

**Legs** (Fig. 7b): appearing dark overall, at least 1/2 of femur yellow, tibia and tarsi yellow.

**Abdomen**(Fig. 7a, b): T1+2 with mid-dorsal depression extending halfway along its length, not reaching tergal margin; median marginal bristles present on T4 and T5 but absent on T1+2 and T3. Discal bristles absent from all tergites. Silver tomentosity on margins of abdominal segmentsT3 and T4 not extending beyond 1/3 of tergal surface.

**Male terminalia** (Fig. 7d, e): cerci tightly juxtaposed when viewed dorsally; haired up to tapering point, then bare until the tip; apparently convex when viewed laterally; very slight thickening apically so as to appear slightly clubbed; surstylus 9/10 the length of the cercus, outwardly convex at its center so as to appear outwardly bowed with a slight inward bend apically, giving it a slightly hooked appearance at its tip, visible in dorsal view; densely bristled along its entire length; phallus 2x as long as cercus, with a downward bend.

#### Diagnosis

*Itaplectops ericpalolai* can be distinguished by the following combination of traits: proclinate orbital bristles present in males; first flagellomere brilliant pale orange; parafacial with 1 bristle arising midway between lowest frontal bristle and facial margin; median marginal bristles absent on T1+2 and T3, but present on T4 and T5; discal bristles absent; silver tomentosity present on margins of abdominal segments T3 and T4. It can be distinguished from its most similar congener, *Itaplectops antennalis*, following couplet 3 in the key to *Itaplectops* (below).

#### Etymology

*Itaplectops ericpalolai* is named in honor of Eric Palola of Vermont, USA, Executive Director of the Guanacaste Dry Forest Conservation Fund, and musician, skier, farmer (complete with chickens) and forester.

#### Distribution

Costa Rica, ACG, Prov. Alajuela, rain forest.

#### Ecology

##### Hosts

Reared from caterpillars of the Dalceridae *Acraga coa* (Schaus, 1892).

### Itaplectops griseobasis sp. n.

urn:lsid:zoobank.org:act:F334625E-1950-4156-B60E-CA3658F8D3F9

#### Materials

- **Type status:** Holotype. **Occurrence:** occurrenceDetails: http://janzen.sas.upenn.edu; catalogNumber: DHJPAR0007073; recordedBy: D.H. Janzen & W. Hallwachs, Gloria Sihezar; individualID: DHJPAR0007073; individualCount: 1; sex: F; lifeStage: adult; preparations: pinned; otherCatalogNumbers: ASTAV315-06, 06-SRNP-2159; **Taxon:** scientificName: Itaplectops griseobasis; phylum: Arthropoda; class: Insecta; order: Diptera; family: Tachinidae; genus: Itaplectops; specificEpithet: griseobasis; scientificNameAuthorship: Fleming & Wood; **Location:** continent: Central America; country: Costa Rica; countryCode: CR; stateProvince: Alajuela; county: Area de Conservacion Guanacaste; locality: Sector San Cristobal; verbatimLocality: Rio Blanco Abajo; verbatimElevation: 500; verbatimLatitude: 10.9; verbatimLongitude: -85.373; verbatimCoordinateSystem: Decimal; decimalLatitude: 10.9; decimalLongitude: -85.373; **Identification:** identifiedBy: AJ Fleming; dateIdentified: 2014; **Event:** samplingProtocol: reared from caterpillar of Euprosterna wemilleri (Limacodidae); verbatimEventDate: 04-Apr-2005; **Record Level:** language: en; institutionCode: CNC; collectionCode: Insects; basisOfRecord: Pinned Specimen
- **Type status:** Paratype. **Occurrence:** occurrenceDetails: http://janzen.sas.upenn.edu; catalogNumber: DHJPAR0030159; recordedBy: D.H. Janzen & W. Hallwachs, Petrona Rios; individualID: DHJPAR0030159; individualCount: 1; sex: F; lifeStage: adult; preparations: pinned; otherCatalogNumbers: ASHYB903-09, 08-SRNP-32765; **Taxon:** scientificName: Itaplectops griseobasis; phylum: Arthropoda; class: Insecta; order: Diptera; family: Tachinidae; genus: Itaplectops; specificEpithet: griseobasis; scientificNameAuthorship: Fleming & Wood; **Location:** continent: Central America; country: Costa Rica; countryCode: CR; stateProvince: Guanacaste; county: Area de Conservacion Guanacaste; locality: Sector Pitilla; verbatimLocality: Pasmompa; verbatimElevation: 440; verbatimLatitude: 11.019; verbatimLongitude: -85.41; verbatimCoordinateSystem: Decimal; decimalLatitude: 11.019; decimalLongitude: -85.41; **Identification:** identifiedBy: AJ Fleming; dateIdentified: 2014; **Event:** samplingProtocol: reared from caterpillar of Natada michorta (Limacodidae); verbatimEventDate: 29-Nov-2008; **Record Level:** language: en; institutionCode: CNC; collectionCode: Insects; basisOfRecord: Pinned Specimen
- **Type status:** Paratype. **Occurrence:** occurrenceDetails: http://janzen.sas.upenn.edu; catalogNumber: DHJPAR0020994; recordedBy: D.H. Janzen & W. Hallwachs, Jose Perez; individualID: DHJPAR0020994; individualCount: 1; sex: F; lifeStage: adult; preparations: pinned; otherCatalogNumbers: ASTA1337-07, 07-SRNP-41902; **Taxon:** scientificName: Itaplectops griseobasis; phylum: Arthropoda; class: Insecta; order: Diptera; family: Tachinidae; genus: Itaplectops; specificEpithet: griseobasis; scientificNameAuthorship: Fleming & Wood; **Location:** continent: Central America; country: Costa Rica; countryCode: CR; stateProvince: Alajuela; county: Area de Conservacion Guanacaste; locality: Sector Rincon Rain Forest; verbatimLocality: Laureles; verbatimElevation: 95; verbatimLatitude: 10.933; verbatimLongitude: -85.253; verbatimCoordinateSystem: Decimal; decimalLatitude: 10.933; decimalLongitude: -85.253; **Identification:** identifiedBy: AJ Fleming; dateIdentified: 2014; **Event:** samplingProtocol: reared from caterpillar of Natada michorta (Limacodidae); verbatimEventDate: 10-Aug-2007; **Record Level:** language: en; institutionCode: CNC; collectionCode: Insects; basisOfRecord: Pinned Specimen
- **Type status:** Paratype. **Occurrence:** occurrenceDetails: http://janzen.sas.upenn.edu; catalogNumber: DHJPAR0023604; recordedBy: D.H. Janzen & W. Hallwachs, Anabelle Cordoba; individualID: DHJPAR0023604; individualCount: 1; sex: F; lifeStage: adult; preparations: pinned; otherCatalogNumbers: ASTAW461-08, 07-SRNP-5030; **Taxon:** scientificName: Itaplectops griseobasis; phylum: Arthropoda; class: Insecta; order: Diptera; family: Tachinidae; genus: Itaplectops; specificEpithet: griseobasis; scientificNameAuthorship: Fleming & Wood; **Location:** continent: Central America; country: Costa Rica; countryCode: CR; stateProvince: Alajuela; county: Area de Conservacion Guanacaste; locality: Sector San Cristobal; verbatimLocality: Puente Palma; verbatimElevation: 460; verbatimLatitude: 10.916; verbatimLongitude: -85.379; verbatimCoordinateSystem: Decimal; decimalLatitude: 10.916; decimalLongitude: -85.379; **Identification:** identifiedBy: AJ Fleming; dateIdentified: 2014; **Event:** samplingProtocol: reared from caterpillar of Natada lalogamezi (Limacodidae); verbatimEventDate: 13-Jan-2008; **Record Level:** language: en; institutionCode: CNC; collectionCode: Insects; basisOfRecord: Pinned Specimen

#### Description

**Male:** unknown at this time.

**Female**

**Length:** 5–7mm.

**Head** (Fig. 8c): proclinate orbital bristles present; first flagellomere entirely dark or brownish orange over at least 1/2 of its surface; arista dark brown over 4/5 of its length, bright orange basally, with gradual taper; first flagellomere reaching facial margin; ocellar bristles reduced, almost hair-like, less than the length of pedicel, arising behind anterior ocellus; ocellar triangle covered in small proclinate hairs; frontal vitta approximately 2x as wide as fronto-orbital plate; facial ridge bare, 4–5 supravibrissal bristles; fronto-orbital plate silver; parafacial entirely silver and bare; fronto-orbital plate with fine bristles not confined to a row and interspersed with frontal bristles, these not extending past lowest frontal bristle.

**Thorax** (Fig. 8a, b): three postsutural supra-alar bristles, anteriormost extremely reduced in size so as to appear hair-like; katepisternum with 2 bristles of equal size, anteriormost arising directly below or slightly in front of suture; apical scutellar bristles short, up to 1/2 length of subapical scutellars; subapical scutellar bristles divergent; scutellum lacking discal bristles.

**Wings** (Fig. 8a): smoky yellow.

**Legs** (Fig. 8b): Appearing light colored overall, femur and tibia yellow, and tarsi haired, appearing black but with yellow ground color; dorso-ventral margin of hind tarsi with yellow tufts of bristles at tarsal apices.

**Abdomen** (Fig. 8a, b): T1+2 with mid-dorsal depression extending halfway along its length, but not reaching tergal margin; median marginal bristles present on all tergites. Discal bristles present on T3, these are shorter than marginal bristles but still obviously bristles. Silver tomentosity entirely covering T1+2 and on anterior margins of abdominal segments T3 and T4, covering up to 1/2 of tergal surface.

#### Diagnosis

*Itaplectops griseobasis* is to date only known from females. It is distinguished by the following combination of traits: first flagellomere brown/black over at least 1/2 of surface; legs yellow with black tarsi; median marginal bristles present on all tergites; discal bristles present at least on T3 (in some cases these can appear reduced but still present); silver tomentosity present on all of T1+2 and on margins of abdominal segments T3, T4, and the underside of T5. It can be distinguished from its most similar congener, *Itaplectops akselpalolai*, following couplet 9 in the key to *Itaplectops* (below).

#### Etymology

From the Latin adjective *grisea*, meaning “gray”, in reference to the silver tomentosity on the entirety of T1+2, and the Latin nound *basis* meaning "foundation".

#### Distribution

Costa Rica, ACG, Prov. Alajuela, rain forest.

#### Ecology

##### Hosts

Reared from caterpillars of 3 species of Limacodidae: *Euprosterna wemilleri* Corrales & Epstein, 2000; *Natada lalogamezi* Corrales, 2000; and *Natada michorta* Dyar, 1912.

### Itaplectops omissus

Fleming & Wood, 2014 sp. n.

urn:lsid:zoobank.org:act:52B9F595-55E4-4EDE-9BE6-31C99EC1C1D8

#### Materials

- **Type status:** Holotype. **Occurrence:** occurrenceDetails: http://janzen.sas.upenn.edu; catalogNumber: DHJPAR0018615; recordedBy: D.H. Janzen & W. Hallwachs, Roster Moraga; individualID: DHJPAR0018615; individualCount: 1; sex: M; lifeStage: adult; preparations: pinned; otherCatalogNumbers: ASTAI1262-07, 97-SRNP-1136; **Taxon:** scientificName: Itaplectops omissus; phylum: Arthropoda; class: Insecta; order: Diptera; family: Tachinidae; genus: Itaplectops; specificEpithet: omissus; scientificNameAuthorship: Fleming & Wood; **Location:** continent: Central America; country: Costa Rica; countryCode: CR; stateProvince: Guanacaste; county: Area de Conservacion Guanacaste; locality: Sector Cacao; verbatimLocality: Estacion Cacao; verbatimElevation: 1150; verbatimLatitude: 10.927; verbatimLongitude: -85.468; verbatimCoordinateSystem: Decimal; decimalLatitude: 10.927; decimalLongitude: -85.468; **Identification:** identifiedBy: AJ Fleming; dateIdentified: 2014; **Event:** samplingProtocol: reared from caterpillar of *Acharia ophelians* (Limacodidae); verbatimEventDate: 26-Sep-2013; **Record Level:** language: en; institutionCode: CNC; collectionCode: Insects; basisOfRecord: Pinned Specimen

#### Description

**Male**

**Length:** 5mm. **Head** (Fig. 9c): proclinate orbital bristles present, although reduced to almost hair-like; first flagellomere entirely dark or brownish orange over at least 1/2 of its surface; arista dark brown over 3/4 of its length, bright orange basally, with gradual taper; first flagellomere reaching facial margin; ocellar bristles reduced, almost hair-like, as long as pedicel, arising behind anterior ocellus; ocellar triangle covered in small proclinate hairs; frontal vitta approximately as wide as fronto-orbital plate; facial ridge bearing at least 4–5 stout, decumbent bristles above vibrissa; fronto-orbital plate slightly gold tinged; parafacial entirely silver and bare; fronto-orbital plate with few fine bristles outside of frontal bristles, these not extending past lowest frontal bristle.

**Thorax** (Fig. 9a, b): three postsutural supra-alar bristles, anteriormost greatly reduced to an almost hair-like structure; katepisternum with 2 bristles, anteriormost reduced in size, arising slightly behind suture; apical scutellar bristles long, up to 3/4 length of subapical scutellars; subapical scutellar bristles parallel or convergent (often crossed); scutellum with no apparent discal bristles.

**Wings** (Fig. 9a, b): smoky gray, with a large dark region surrounding the anterior portions of vein R_2+3 _and R_4+5_ as they approach the wing margin.

**Legs** (Fig. 9b): Appearing dark overall, femur at least 1/2 yellow, tibia yellow, and tarsi dark; dorso-ventral margin of hind tarsi with yellow tufts of bristles apically.

**Abdomen** (Fig. 9a, b): T1+2 with mid-dorsal depression extending 2/3 along its length, but not reaching tergal margin; median marginal bristles present on all tergites except T1+2. Reduced discal bristles present on T3; these can have the appearance of thicker abdominal hairs. Silver tomentosity present on margins of abdominal segments T3 and T4, only visible under certain angles of light, with these not extending beyond 1/3 of tergal surface.

**Terminalia** (Fig. 9d, e): cerci tightly juxtaposed basally and diverging at the tip; haired up to tapering point, after which they becomes bare until tip; cercus apparently convex when viewed laterally; with a very slight thickening apically so as to appear slightly clubbed; surstylus as long as cercus, outwardly convex with a strong inward curve at its center, giving it a slightly hooked tip, due to the nature of the curvature this character is most visible from above in this species; densely bristled along its entire length; phallus 2.5x as long as cercus, with a downward bend.

**Female:** unknown at this time.

#### Diagnosis

*Itaplectops omissus* can be distinguished by the following combination of traits: proclinate orbital bristles present in male although these appear to be extremely reduced, appearing as just fronto-orbital hairs; first flagellomere black/brown over 1/2 of surface; femur at least 1/2 yellow, tibia yellow, and tarsi dark; median marginal bristles absent on T1+2 but present on T3, T4 and T5; discal bristles present at least on T3 (in some cases these can appear reduced but still present); silver tomentosity present on margins of abdominal segments T3 and T4. It can be distinguished from its most similar congeners, *Itaplectops griseobasis* and *I. akselpalolai*, following couplet 8 in the key to *Itaplectops* (below).

#### Etymology

From the Latin participle of the verb “*omitto*”, meaning to leave out or omit, referring to the lack of median marginals on T1+2.

#### Distribution

Costa Rica, ACG, Prov. Guanacaste, cloud forest.

#### Ecology

##### Hosts

Reared from caterpillars of the Limacodidae, *Acharia ophelians* (Dyar, 1927).

### Itaplectops shellymcsweeneyae

Fleming & Wood, 2014 sp. n.

urn:lsid:zoobank.org:act:5B915E15-C94A-4410-99AB-7DC2CAE14A27

#### Materials

- **Type status:** Holotype. **Occurrence:** occurrenceDetails: http://janzen.sas.upenn.edu; catalogNumber: DHJPAR0016540; recordedBy: D.H. Janzen & W. Hallwachs, Manuel Rios; individualID: DHJPAR0016540; individualCount: 1; sex: M; lifeStage: adult; preparations: pinned; otherCatalogNumbers: ASTAP744-07, 06-SRNP-65647; **Taxon:** scientificName: Itaplectops shellymcsweeneyae; phylum: Arthropoda; class: Insecta; order: Diptera; family: Tachinidae; genus: Itaplectops; specificEpithet: shellymcsweeneyae; scientificNameAuthorship: Fleming & Wood; **Location:** continent: Central America; country: Costa Rica; countryCode: CR; stateProvince: Guanacaste; county: Area de Conservacion Guanacaste; locality: Sector Pitilla; verbatimLocality: Sendero Laguna; verbatimElevation: 680; verbatimLatitude: 10.989; verbatimLongitude: -85.423; verbatimCoordinateSystem: Decimal; decimalLatitude: 10.989; decimalLongitude: -85.423; **Identification:** identifiedBy: AJ Fleming; dateIdentified: 2014; **Event:** samplingProtocol: reared from caterpillar of *Venadicodia caneti* (Limacodidae); verbatimEventDate: 11-Jan-2007; **Record Level:** language: en; institutionCode: CNC; collectionCode: Insects; basisOfRecord: Pinned Specimen
- **Type status:** Paratype. **Occurrence:** occurrenceDetails: http://janzen.sas.upenn.edu; catalogNumber: DHJPAR0029577; recordedBy: D.H. Janzen & W. Hallwachs, Jose Cortez; individualID: DHJPAR0029577; individualCount: 1; sex: F; lifeStage: adult; preparations: pinned; otherCatalogNumbers: ASHYM998-09, 08-SRNP-57655; **Taxon:** scientificName: Itaplectops shellymcsweeneyae; phylum: Arthropoda; class: Insecta; order: Diptera; family: Tachinidae; genus: Itaplectops; specificEpithet: shellymcsweeneyae; scientificNameAuthorship: Fleming & Wood; **Location:** continent: Central America; country: Costa Rica; countryCode: CR; stateProvince: Guanacaste; county: Area de Conservacion Guanacaste; locality: Sector Mundo Nuevo; verbatimLocality: Camino Pozo Tres; verbatimElevation: 733; verbatimLatitude: 10.771; verbatimLongitude: -85.374; verbatimCoordinateSystem: Decimal; decimalLatitude: 10.771; decimalLongitude: -85.374; **Identification:** identifiedBy: AJ Fleming; dateIdentified: 2014; **Event:** samplingProtocol: reared from caterpillar of *Venadicodia caneti* (Limacodidae); verbatimEventDate: 11-Oct-2008; **Record Level:** language: en; institutionCode: CNC; collectionCode: Insects; basisOfRecord: Pinned Specimen
- **Type status:** Paratype. **Occurrence:** occurrenceDetails: http://janzen.sas.upenn.edu; catalogNumber: DHJPAR0027821; recordedBy: D.H. Janzen & W. Hallwachs, Mariano Pereira; individualID: DHJPAR0027821; individualCount: 1; sex: M; lifeStage: adult; preparations: pinned; otherCatalogNumbers: ASHYE058-08, 08-SRNP-57048; **Taxon:** scientificName: Itaplectops shellymcsweeneyae; phylum: Arthropoda; class: Insecta; order: Diptera; family: Tachinidae; genus: Itaplectops; specificEpithet: shellymcsweeneyae; scientificNameAuthorship: Fleming & Wood; **Location:** continent: Central America; country: Costa Rica; countryCode: CR; stateProvince: Guanacaste; county: Area de Conservacion Guanacaste; locality: Sector Mundo Nuevo; verbatimLocality: Porton Rivas; verbatimElevation: 570; verbatimLatitude: 10.759; verbatimLongitude: -85.373; verbatimCoordinateSystem: Decimal; decimalLatitude: 10.759; decimalLongitude: -85.373; **Identification:** identifiedBy: AJ Fleming; dateIdentified: 2014; **Event:** samplingProtocol: reared from caterpillar of *Venadicodia caneti* (Limacodidae); verbatimEventDate: 21-Aug-2008; **Record Level:** language: en; institutionCode: CNC; collectionCode: Insects; basisOfRecord: Pinned Specimen

#### Description

**Male and female**

**Length:** male 5–6mm; female 6mm.

**Head** (Fig. 10c): proclinate orbital bristles present in both males and females; first flagellomere brilliant pale orange; arista brilliant pale orange at its base and darkening to brown at its tip, with gradual taper; first flagellomere slightly shorter than facial margin by a distance not exceeding the length of the pedicel; ocellar bristles reduced, almost hair-like, no longer than length of pedicel, arising behind anterior ocellus; ocellar triangle bare; frontal vitta not quite 2x as wide as fronto-orbital plate; facial ridge bearing 4 stout, decumbent bristles; fronto-orbital plate and parafacial entirely silver and bare; fronto-orbital plate of male with one row of very fine bristles lateral to frontal bristles, these not extending past upper margin of pedicel; absent in female.

**Thorax** (Fig. 10a): three postsutural supra-alar bristles, anteriormost greatly reduced to an almost hair-like structure; katepisternum with 2 bristles, anteriormost arising slightly behind suture; apical scutellar bristles short, up to 1/2 length of subapical scutellars; subapical scutellar bristles parallel or convergent (often crossed); scutellum with 1 or 2 pairs of widely separated discal bristles.

**Wings** (Fig. 10a): smoky yellow.

**Legs** (Fig. 10b): Appearing light overall, femur at least 1/2 yellow, tibia yellow, and tarsi darkened dueto densely haired surface; dorso-ventral margin of hind tarsi with yellow tufts of bristles apically.

**Abdomen** (Fig. 10a, b): T1+2 with mid-dorsal depression extending over2/3 of its length, but not reaching tergal margin; median marginal bristles present on T3, T4 and T5 but absent on T1+2. Discal bristles absent. Silver tomentosity present on margins of abdominal segments T3, T4, and T5 but not extending beyond 1/3 of tergal surface.

**Male terminalia** (Fig. 10d, e): both cerci tightly juxtaposed (neither fused nor diverging) when viewed dorsally; haired up to tapering point, after which they become bare until the tip, which is strongly clubbed; apparently convex when viewed laterally; surstylus 9/10 the length of the cercus, not outwardly convex at its center, hooked downward at its tip; sparsely bristled along its entire length; phallus 1.5x as long as cercus, with a downward bend.

#### Diagnosis

*Itaplectops shellymcsweeneyae* can be distinguished by the following combination of traits: proclinate orbital bristles present in male; first flagellomere brilliant pale orange; median marginal bristles present on T3, T4 and T5 but absent on T1+2; discal bristles absent from all tergites; silver tomentosity present on margins of abdominal segments T3, T4, and T5. It can be distinguished from its most similar congeners, *Itaplectops antennalis* and *I. ericpalolai*, following couplet 2 in the key to *Itaplectops* (below).

#### Etymology

*Itaplectops shellymcsweeneyae* is named in honor of Shelly McSweeney of Vermont, USA, a crucial supporter of the Palola-McSweeney family, the Guanacaste Dry Forest Conservation Fund, and therefore ACG.

#### Distribution

Costa Rica, ACG, Prov. Guanacaste, rain forest.

#### Ecology

##### Hosts

Reared from caterpillars of the Limacodidae *Venadicodia caneti* Corralles & Epstein, 1995.

### Itaplectops tristanpalolai

Fleming & Wood, 2014 sp. n.

urn:lsid:zoobank.org:act:79845A2C-0AA4-4445-B258-573F740830D3

#### Materials

- **Type status:** Holotype. **Occurrence:** occurrenceDetails: http://janzen.sas.upenn.edu; catalogNumber: DHJPAR0011743; recordedBy: D.H. Janzen & W. Hallwachs, Manuel Rios; individualID: DHJPAR0011743; individualCount: 1; sex: M; lifeStage: adult; preparations: pinned; otherCatalogNumbers: ASTAS469-06, 05-SRNP-33719; **Taxon:** scientificName: Itaplectops tristanpalolai; phylum: Arthropoda; class: Insecta; order: Diptera; family: Tachinidae; genus: Itaplectops; specificEpithet: tristanpalolai; scientificNameAuthorship: Fleming & Wood; **Location:** continent: Central America; country: Costa Rica; countryCode: CR; stateProvince: Guanacaste; county: Area de Conservacion Guanacaste; locality: Sector Pitilla; verbatimLocality: Pasmompa; verbatimElevation: 440; verbatimLatitude: 11.019; verbatimLongitude: -85.41; verbatimCoordinateSystem: Decimal; decimalLatitude: 11.019; decimalLongitude: -85.41; **Identification:** identifiedBy: AJ Fleming; dateIdentified: 2014; **Event:** samplingProtocol: reared from caterpillar of *Epiperola paida* (Limacodidae); verbatimEventDate: 03-Oct-2005; **Record Level:** language: en; institutionCode: CNC; collectionCode: Insects; basisOfRecord: Pinned Specimen
- **Type status:** Paratype. **Occurrence:** occurrenceDetails: http://janzen.sas.upenn.edu; catalogNumber: DHJPAR0011742; recordedBy: D.H. Janzen & W. Hallwachs, Manuel Rios; individualID: DHJPAR0011742; individualCount: 1; sex: F; lifeStage: adult; preparations: pinned; otherCatalogNumbers: ASTAS468-06, 05-SRNP-33720; **Taxon:** scientificName: Itaplectops tristanpalolai; phylum: Arthropoda; class: Insecta; order: Diptera; family: Tachinidae; genus: Itaplectops; specificEpithet: tristanpalolai; scientificNameAuthorship: Fleming & Wood; **Location:** continent: Central America; country: Costa Rica; countryCode: CR; stateProvince: Guanacaste; county: Area de Conservacion Guanacaste; locality: Sector Pitilla; verbatimLocality: Pasmompa; verbatimElevation: 440; verbatimLatitude: 11.019; verbatimLongitude: -85.41; verbatimCoordinateSystem: Decimal; decimalLatitude: 11.019; decimalLongitude: -85.41; **Identification:** identifiedBy: AJ Fleming; dateIdentified: 2014; **Event:** samplingProtocol: reared from caterpillar of *Epiperola paida* (Limacodidae); verbatimEventDate: 25-Sep-2005; **Record Level:** language: en; institutionCode: CNC; collectionCode: Insects; basisOfRecord: Pinned Specimen

#### Description

**Male and female**

**Length:** male 6mm; female 6mm.

**Head** (Fig. 11c): proclinate orbital bristles present in both male and female; first flagellomere entirely dark or brownish orange over at least 1/2 of its surface; arista dark brown over 3/4 of its length, with gradual taper; frontal vitta bearing a gold sheen when viewed from the front; first flagellomere reaching facial margin; ocellar bristles reduced, almost hair-like, no longer than length of pedicel, arising between posterior ocelli; ocellar triangle bare; frontal vitta approximately 2x as wide as fronto-orbital plate; facial ridge bearing 5–6 stout decumbent bristles; fronto-orbital plate and parafacial entirely silver; parafacial mostly bare though with a few randomly placed fine bristles; fronto-orbital plate of male with fine bristles confined to a row lateral to frontal bristles, these not extending past upper lowest frontal bristl; absent in female.

**Thorax** (Fig. 11a, b): three postsutural supra-alar bristles, anteriormost greatly reduced to an almost hair-like structure; katepisternum with 2 bristles, anteriormost reduced in size, arising slightly behind suture; apical scutellar bristles long, up to 3/4 length of subapical scutellars; subapical scutellar bristles parallel or convergent (often crossed); scutellum with 1 or 2 pairs of widely separated discal bristles.

**Wings** (Fig. 11a): smoky yellow.

**Legs** (Fig. 11b): appearing dark overall, femur at least 1/2 yellow, tibia yellow, and tarsi yellow (although these appear dark due to hirsuteness); dorso-ventral margin of hind tarsi lacking yellow tufts of bristles apically.

**Abdomen** (Fig. 11a, b): T1+2 with mid-dorsal depression extending halfway along its length, not reaching tergal margin; median marginal bristles present on T4 and T5 but absent on T1+2 and T3. Discal bristles absent. Silver tomentosity present on margins of abdominal segments T3 and T4, only visible under certain angles and not extending beyond 1/3 of tergal surface.

**Male terminalia** (Fig. 11d, e): both cerci tightly juxtaposed basally, diverging at their tips; cercus haired up to 3/4 of its length; apparently convex when viewed laterally; surstylus 9/10 the length of the cercus, outwardly convex at its center so as to appear slightly outwardly bowed, in lateral view cercus, appears downwardly curved apically giving it a very slight hook at its tip; short stout bristles present along its entire length; phallus 2x as long as cercus, with a downward bend.

#### Diagnosis

*Itaplectops tristanpalolai* can be distinguished by the following combination of traits: proclinate orbital bristles present in males; first flagellomere brown/black over 1/2 of surface; legs entirely yellow; median marginal bristles absent on T1+2, absent on T3, but present on T4 and T5; discal bristles absent from all tergites; silver tomentosity present on margins of abdominal segments T3 and T4. It can be distinguished from its most similar congener, *Itaplectops anikenpalolae*, following couplet 5 in the key to *Itaplectops* (below).

#### Etymology

*Itaplectops tristanpalolai* is named in honor of Tristan Palola of Vermont, USA, a supporter of Eric Palola and Shelly McSweeney, and therefore of GDFCF and ACG.

#### Distribution

Costa Rica, ACG, Prov. Guanacaste, rain forest and dry forest.

#### Ecology

##### Hosts

Reared from caterpillars of the Limacodidae *Epiperola paida* Dyar, 1912.

## Identification Keys

### Modified key to the genera of Uramyini

**Table:** 

1	Abdominal T5 usually extended dorsally beyond the genitalia, into a tail-like process; proclinate orbital bristles only present in females; T3, T4, and T5 with two or three, rarely one pair of median discal bristles; if no tail present then mid-dorsal depression on T1+2 extending almost to tergal margin	*Uramya* Robineau-Desvoidy
–	Abdominal T5 normal, dorsally with a truncate appearance; two strong proclinate orbital bristles; inner and outer vertical bristles strong, median discal bristles ranging from absent to only one pair present; mid-dorsal depression on T1+2 not extending to tergal margin	2
2	Three katepisternal bristles; discal bristles absent on scutellum; discal bristles present on T4	*Thelairaporia* Townsend
–	Two katepisternal bristles; discal bristles present on scutellum; discal bristles absent on T4	*Itaplectops* Townsend

This key has been adapted from Guimarães (1980) to include the genus *Itaplectops* Townsend; this key is valid for both sexes.

### Key to *Itaplectops*

**Table:** 

1	First flagellomere pale orange	2
–	First flagellomere a darker orange brown	4
2	Median marginal bristles present on T3; apical scutellar bristles short, up to 1/2 the length of subapical scutellars; femur mostly dark, paler at apex (concolorous with tibia)	*I. shellymcsweeneyae* **sp. nov.**
–	Median marginal bristles absent on T3; apical scutellar bristles long at least 1/2 the length of subapical scutellars; femur mostly yellow, darkened at base	3
3	Parafacial with 1 bristle arising near midway, between lowest frontal bristle and facial margin	*I. antennalis* Townsend, 1927
–	Parafacial bare	*I. ericpalolai* **sp. nov.**
4	Parafacial haired, bearing at least a few sparse bristles midway	5
–	Parafacial bare, devoid of any bristles below lowest frontal bristles	6
5	Fronto-orbital plate with small hairs interspersed throughout and not confined to a row outside of frontal bristles, hairs extending into frontal vitta; frontal vitta with silver sheen when viewed from the front; legs dark with yellow ground color on joints, yellow tibia and dark tarsi.	*I. anikenpalolae* **sp. nov.**
–	Fronto-orbital plate with small hairs confined to a row outside of frontal bristles, hairs not extending into frontal vitta; frontal vitta bearing a gold sheen when viewed from the front; legs dark with yellow ground color on joints, yellow tibia and yellow tarsi	*I. tristanpalolai* **sp. nov.**
6	Subapical scutellar bristles either parallel or convergent (frequently crossed); anterior katepisternal arising behind pleural suture; median marginal bristles absent on T1+2, discal bristles absent	7
–	Subapical scutellar bristles divergent; anterior katepisternal arising below or slightly anterior to pleural suture; median marginal bristles present or absent on T1+2 discal bristles present at least on T3	8
7	Fronto-orbital plate entirely golden tinged; facial ridge bearing at least 8 stout decumbent bristles	*I. aurifrons* **sp. nov.**
–	Fronto-orbital plate entirely silver; facial ridge bearing at most 4 stout decumbent bristles	*I. argentifrons* **sp. nov.**
8	Median marginal bristles absent on T1+2; 3 postsutural supra-alar bristles; apical scutellar bristles short, up to 1/2 as long as subapical scutellars; femur yellow, tibia yellow, and dark tarsi	*I. omissus* **sp. nov.**
–	Median marginal bristles present on T1+2; 3 postsutural supra-alar bristles	9
9	Abdominal T1+2 entirely silver tomentose	*I. griseobasis* ****sp. nov.****
–	Abdominal T1+2 with silver tomentosity confined to anterior 1/3 of tergite	*I. akselpalolai* **sp. nov.**

## Supplementary Material

Supplementary material 1 NJ Tree Itaplectops Nov 2015 Data type: Neighbor-Joining tree
Brief description: Neighbor-joining tree of ACG *Itaplectops* as of January, 2015. The inventory is ongoing, and as new specimens are processed these will be added to the database and accessible in BOLD.
File: oo_63251.pdf Fleming et al.

XML Treatment for Itaplectops akselpalolai

XML Treatment for Itaplectops anikenpalolae

XML Treatment for Itaplectops antennalis

XML Treatment for Itaplectops argentifrons

XML Treatment for Itaplectops aurifrons

XML Treatment for Itaplectops ericpalolai

XML Treatment for Itaplectops griseobasis

XML Treatment for Itaplectops omissus

XML Treatment for Itaplectops shellymcsweeneyae

XML Treatment for Itaplectops tristanpalolai

## Figures and Tables

**Figure 2a.**
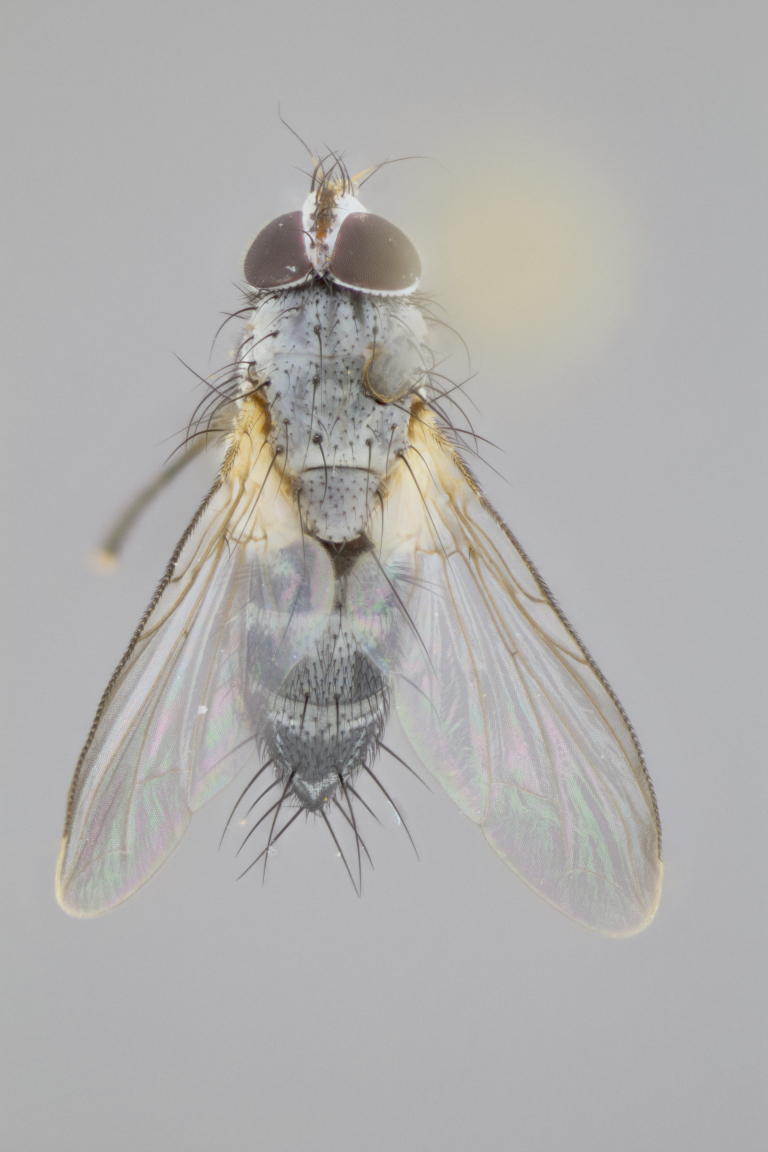
dorsal habitus

**Figure 2b.**
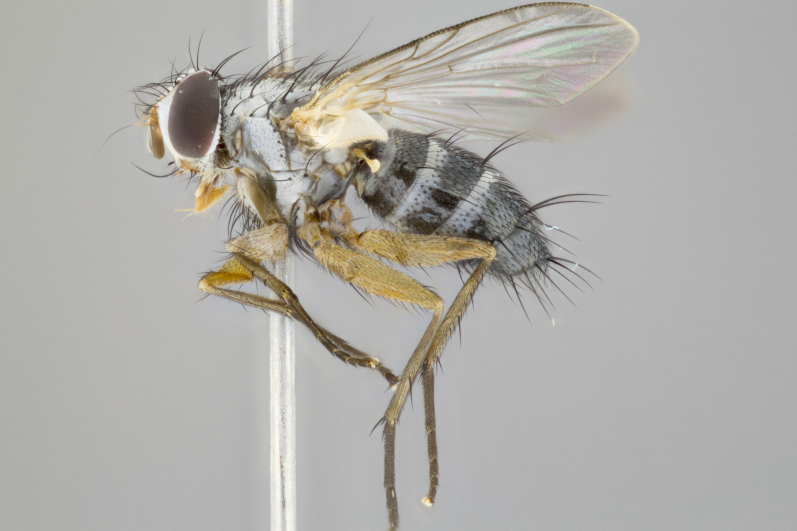
lateral habitus

**Figure 2c.**
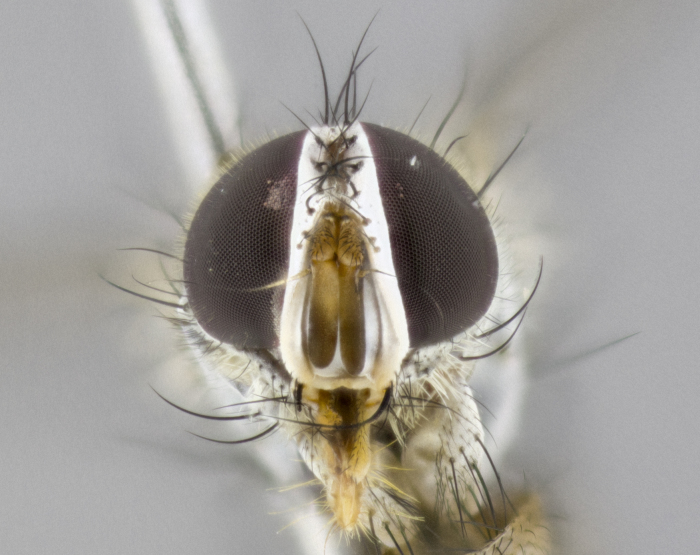
frontal view of head

**Figure 2d.**
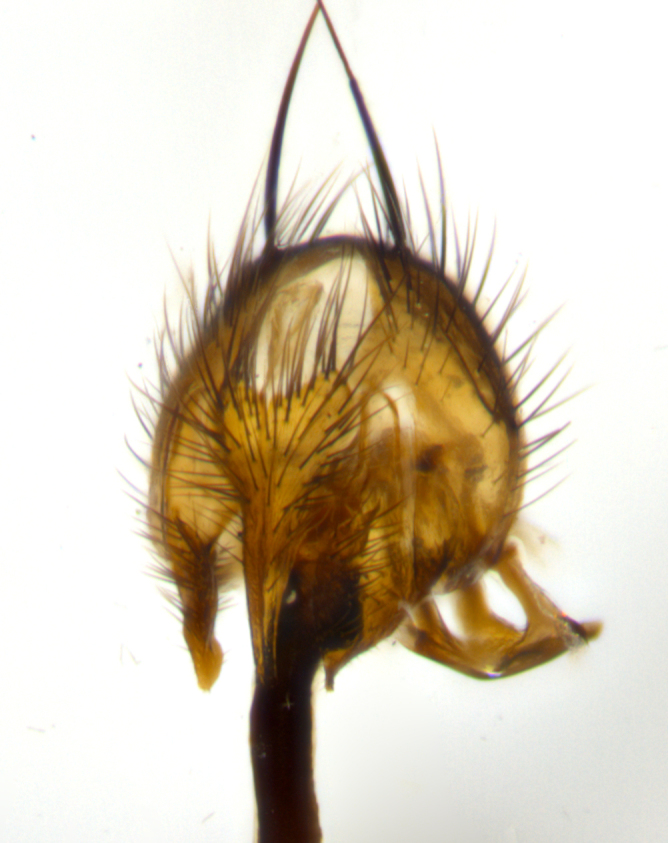
dorsal view of terminalia

**Figure 2e.**
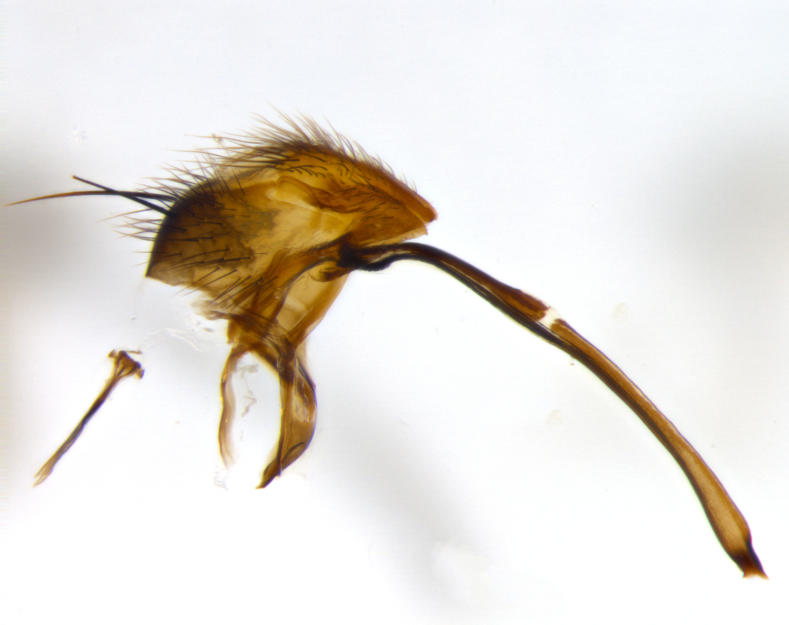
lateral view of terminalia

**Figure 3a.**
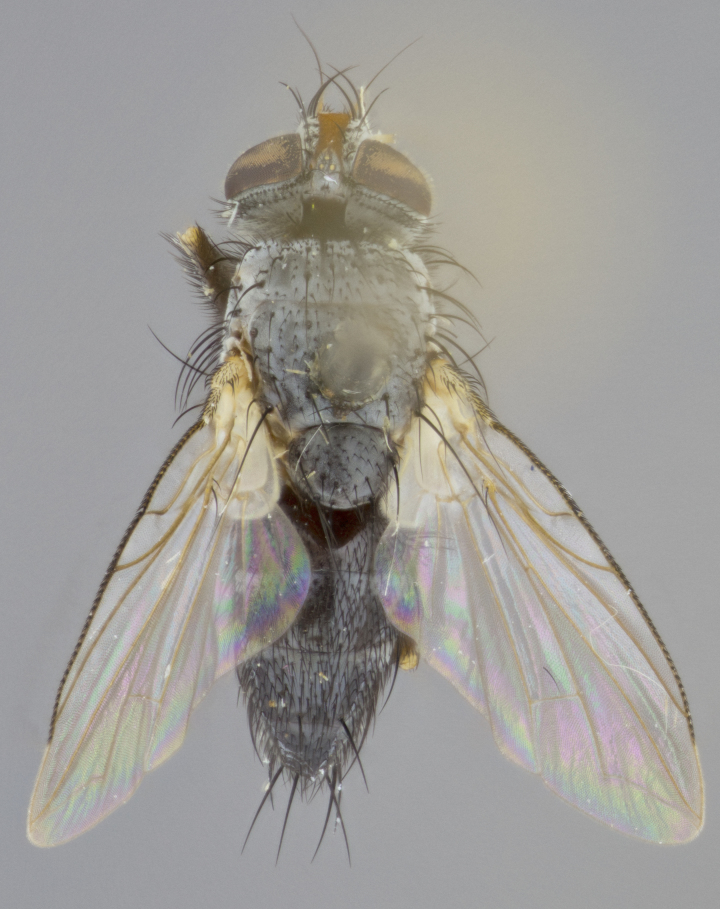
dorsal habitus

**Figure 3b.**
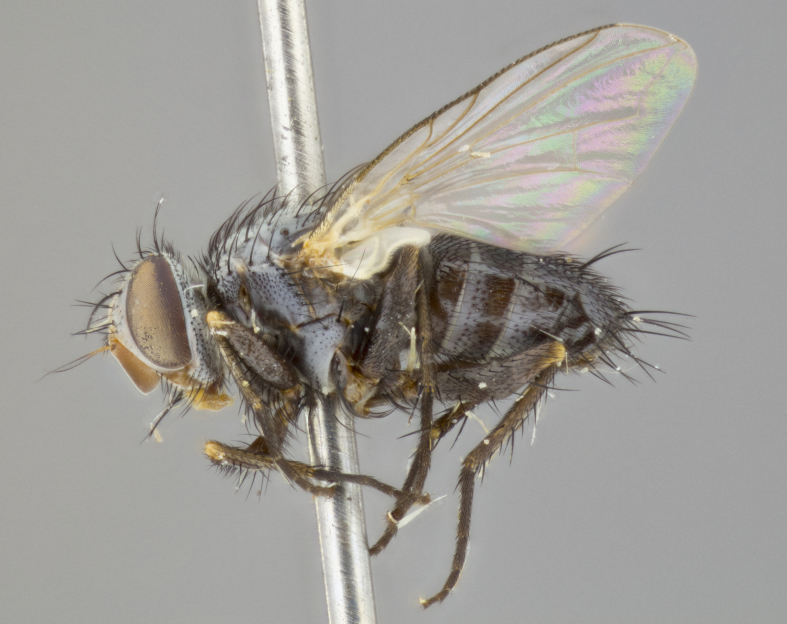
lateral habitus

**Figure 3c.**
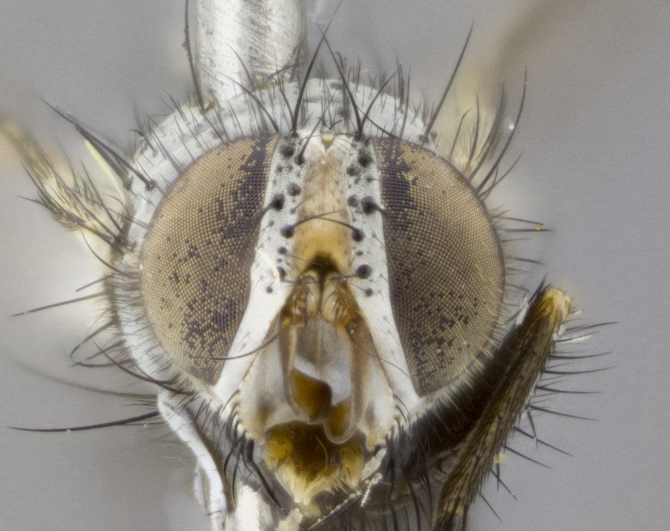
frontal view of head

**Figure 3d.**
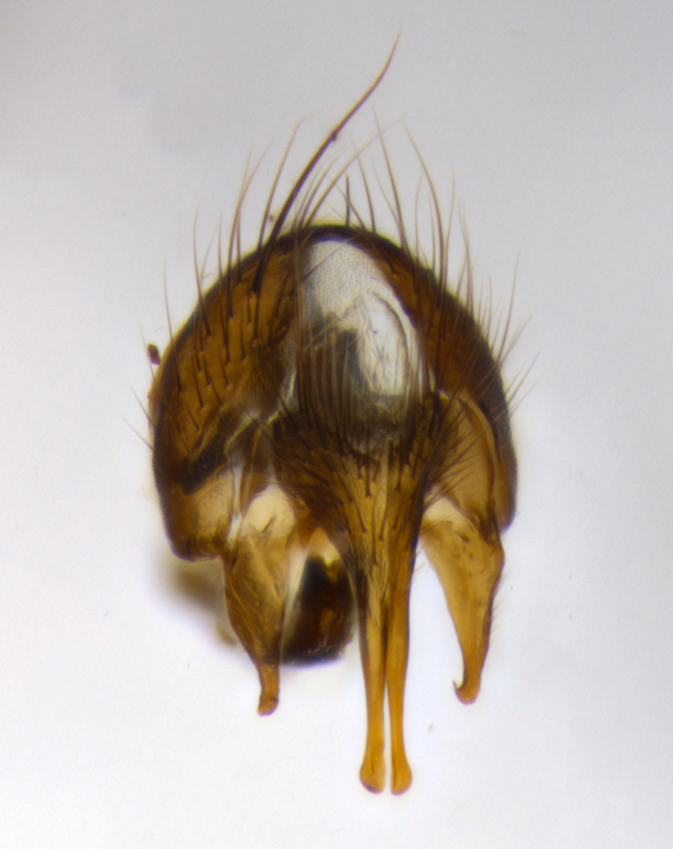
dorsal view of terminalia

**Figure 3e.**
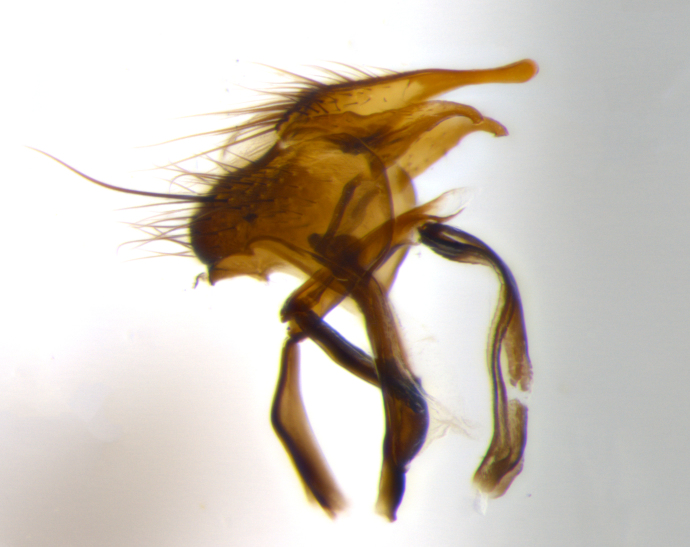
lateral view of terminalia

**Figure 4a.**
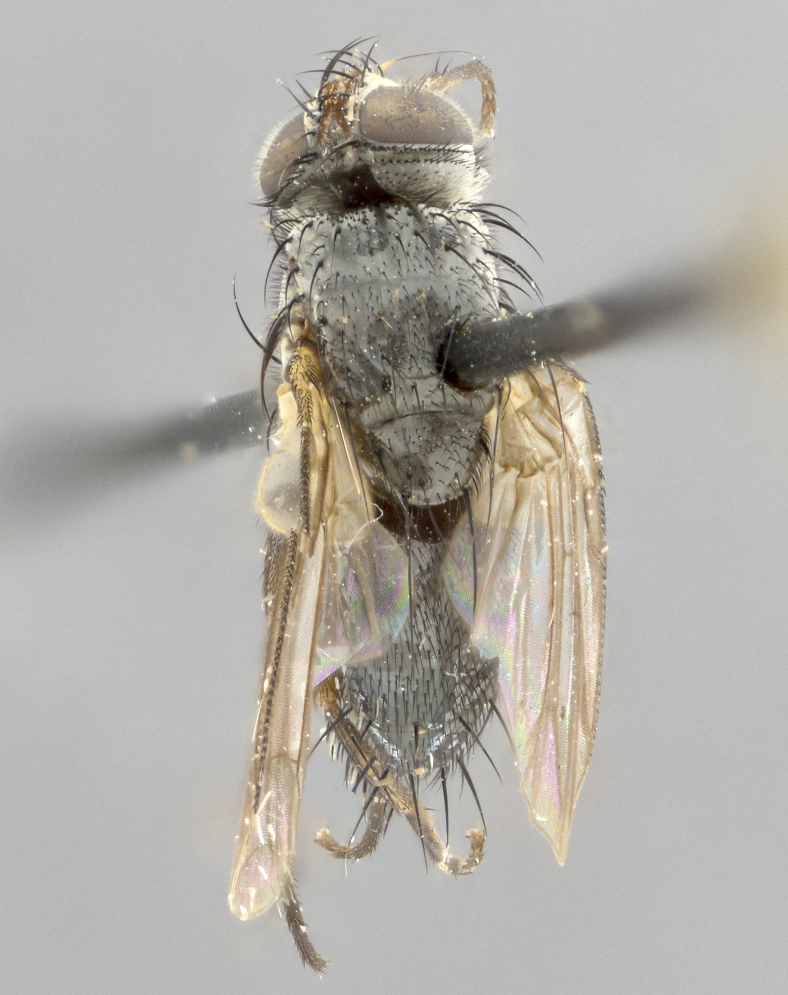
dorsal habitus

**Figure 4b.**
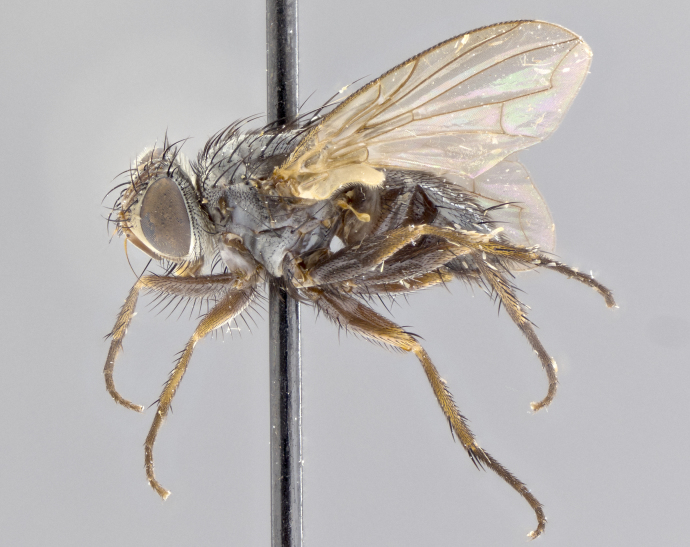
lateral habitus

**Figure 4c.**
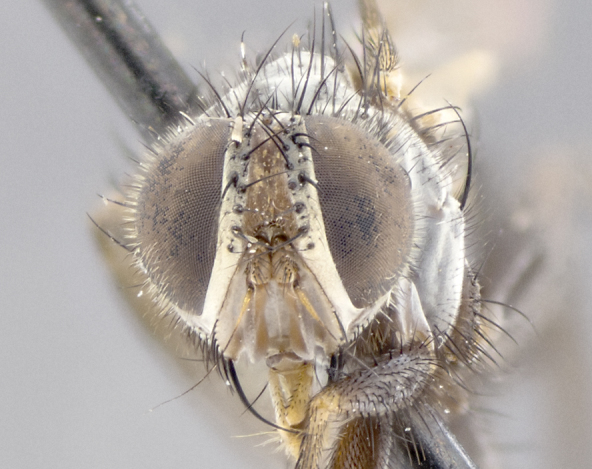
frontal view of head

**Figure 5a.**
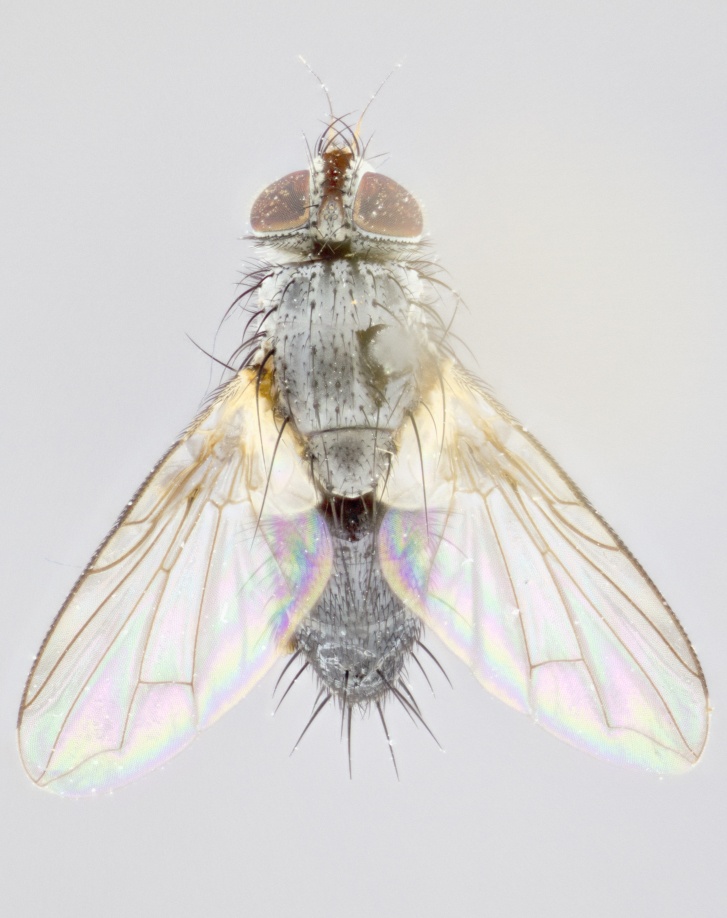
dorsal habitus

**Figure 5b.**
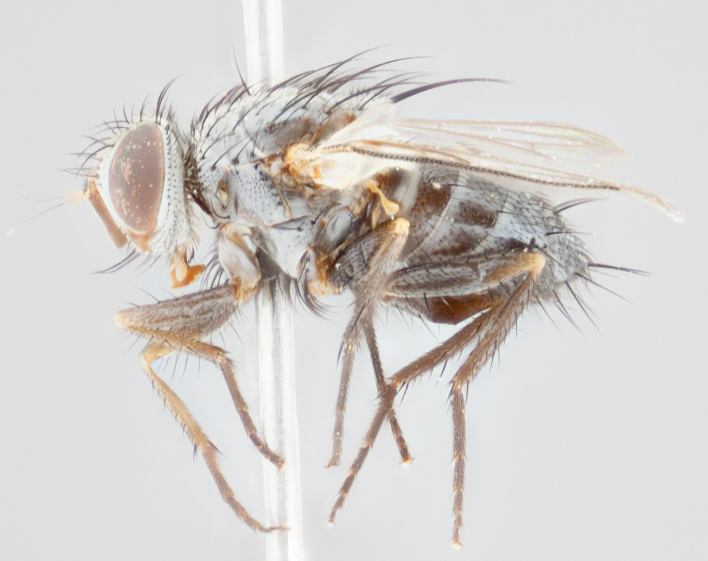
lateral habitus

**Figure 5c.**
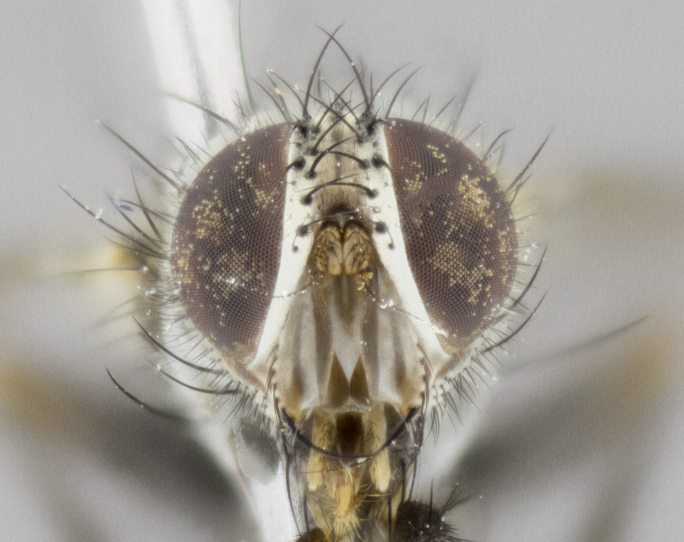
frontal view of head

**Figure 5d.**
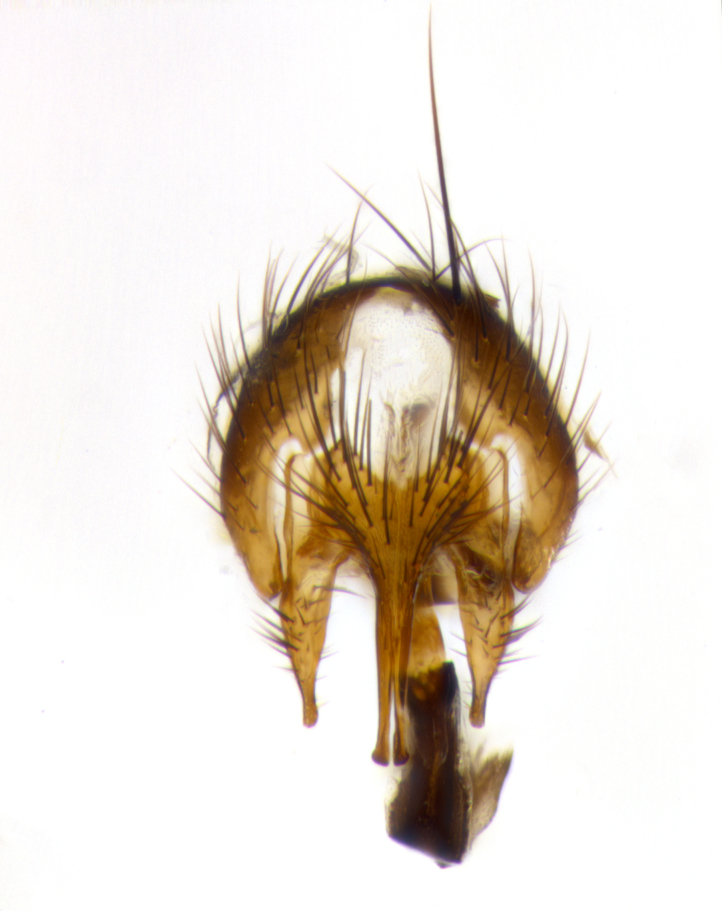
dorsal view of terminalia

**Figure 5e.**
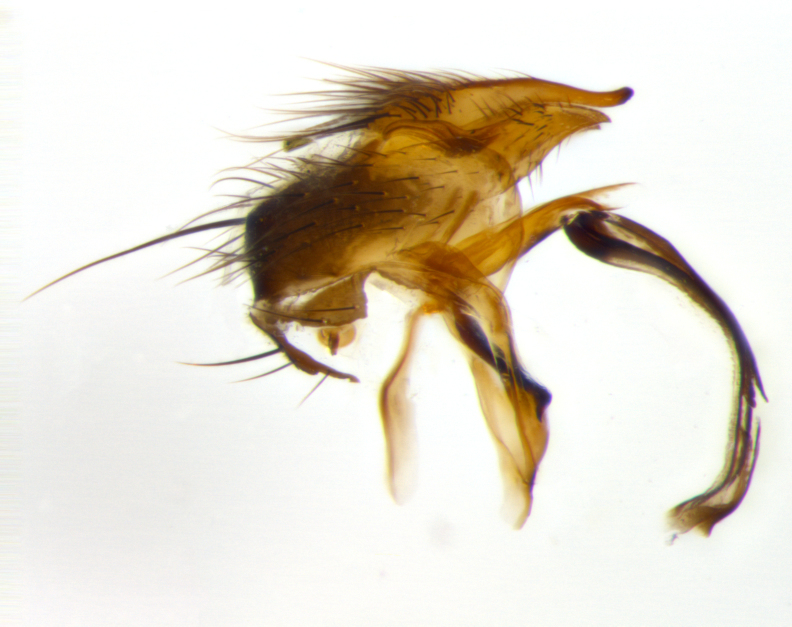
lateral view of terminalia

**Figure 6a.**
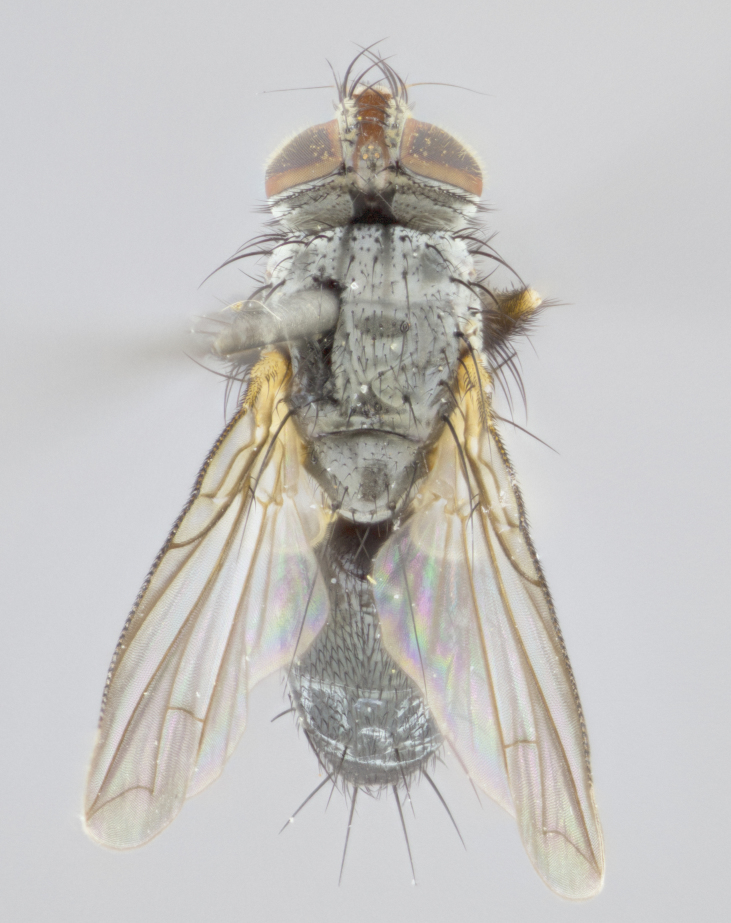
dorsal habitus

**Figure 6b.**
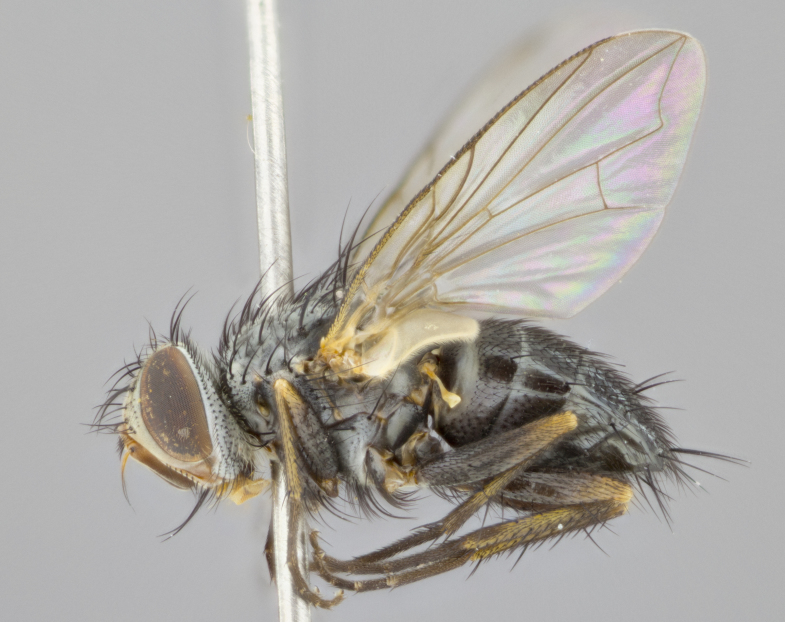
lateral habitus

**Figure 6c.**
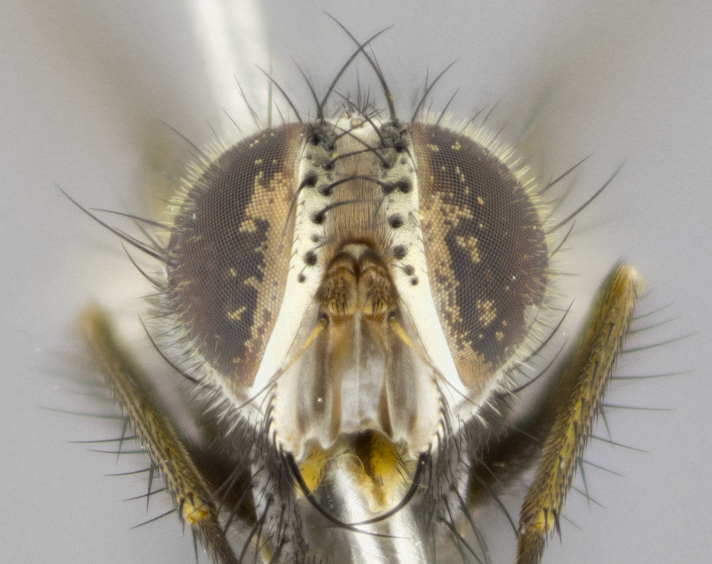
frontal view of head

**Figure 6d.**
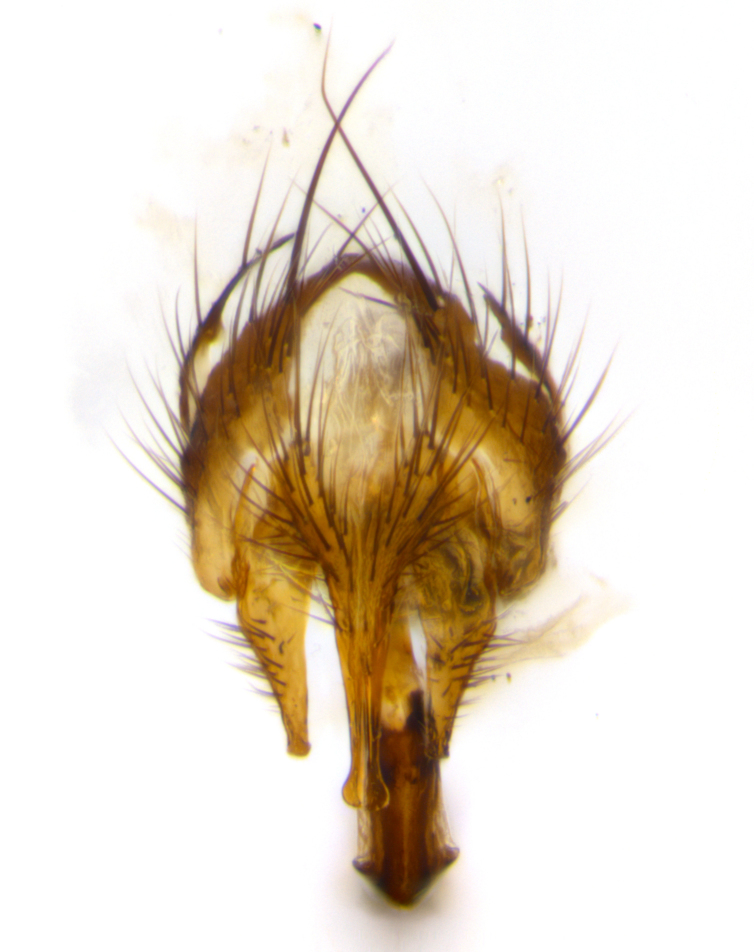
dorsal view of terminalia

**Figure 6e.**
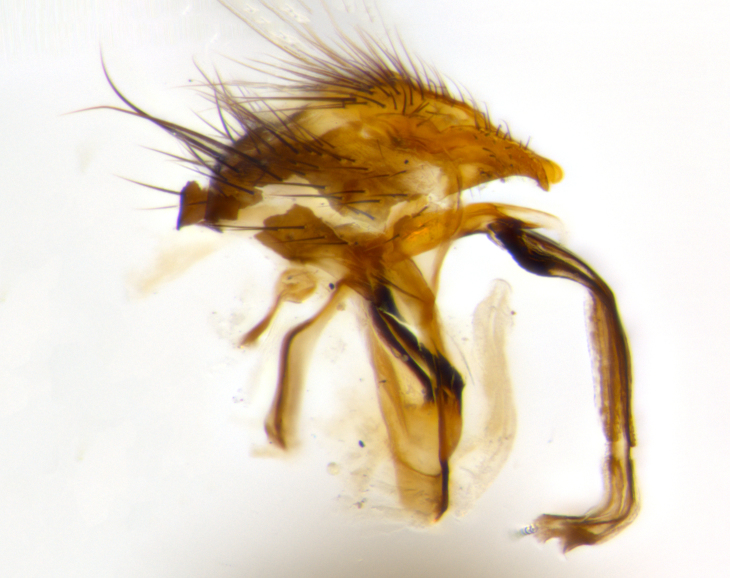
lateral view of terminalia

**Figure 7a.**
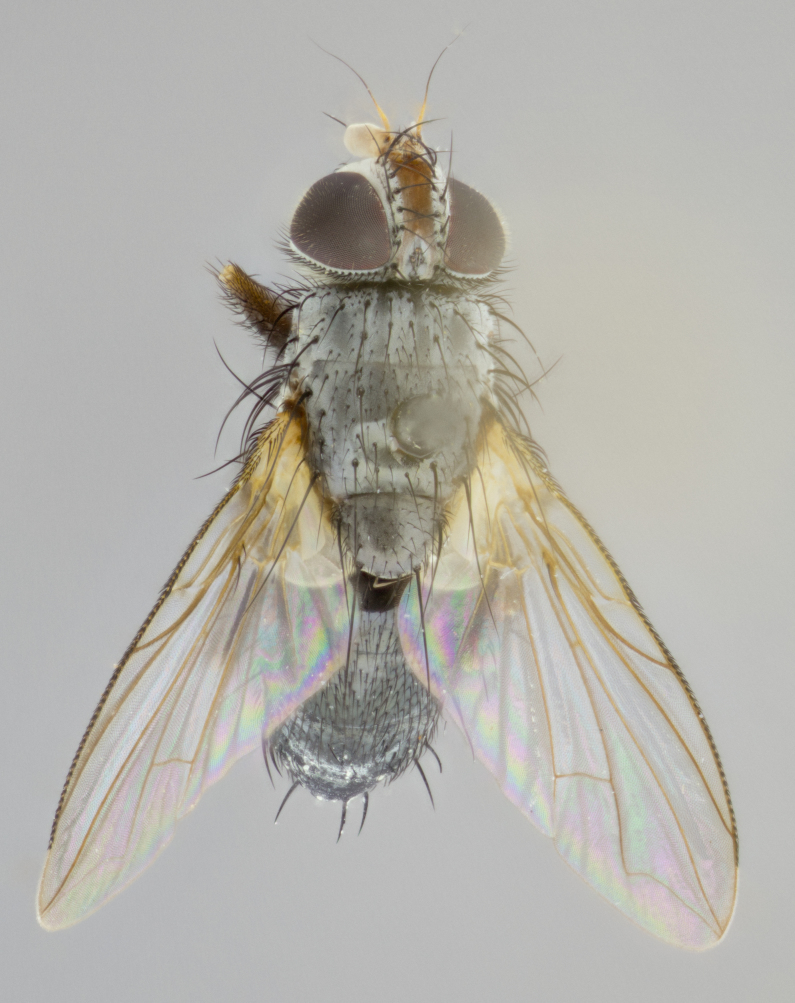
dorsal habitus

**Figure 7b.**
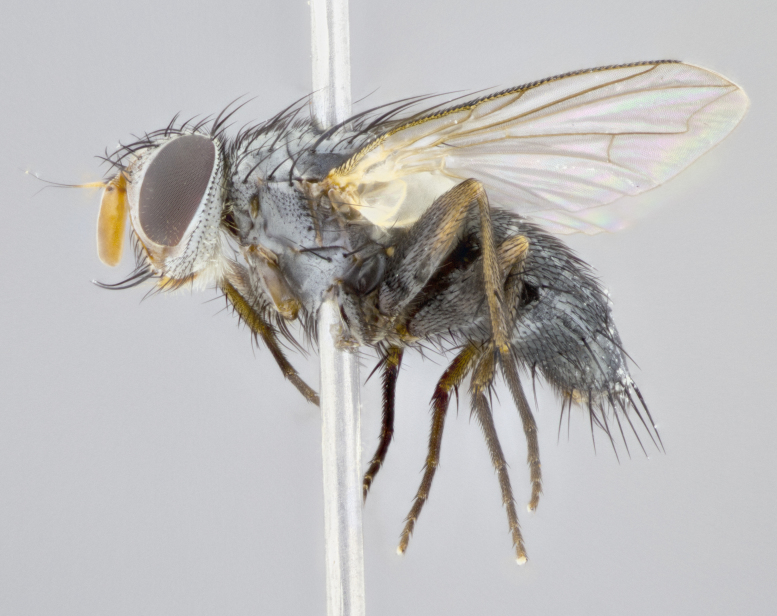
lateral habitus

**Figure 7c.**
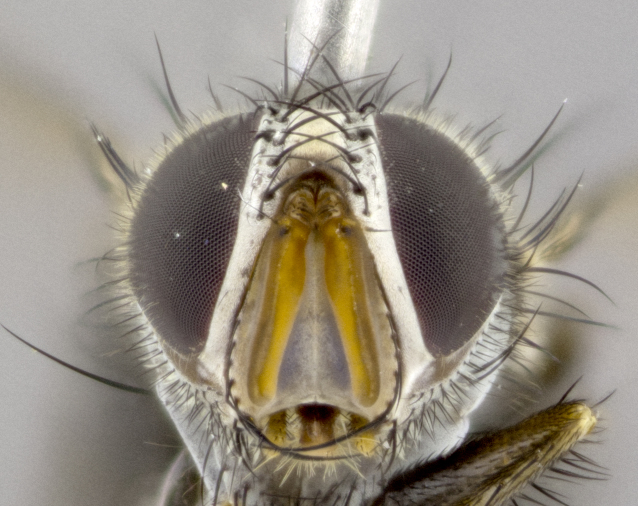
frontal view of head

**Figure 7d.**
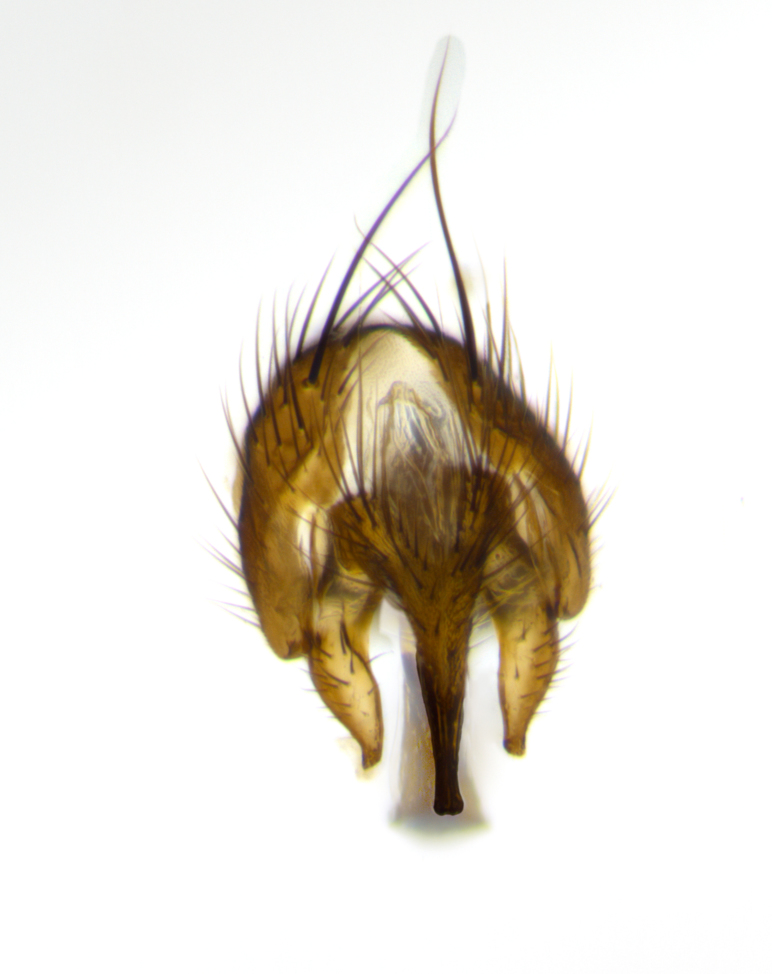
dorsal view of terminalia

**Figure 7e.**
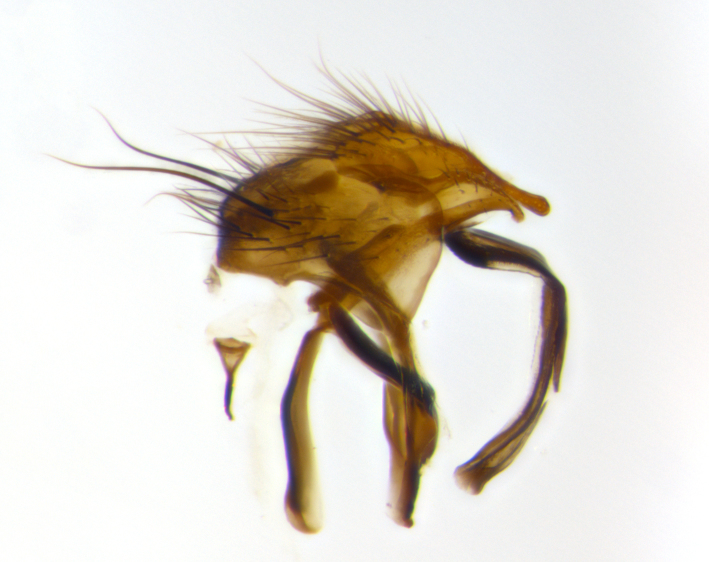
lateral view of terminalia

**Figure 8a.**
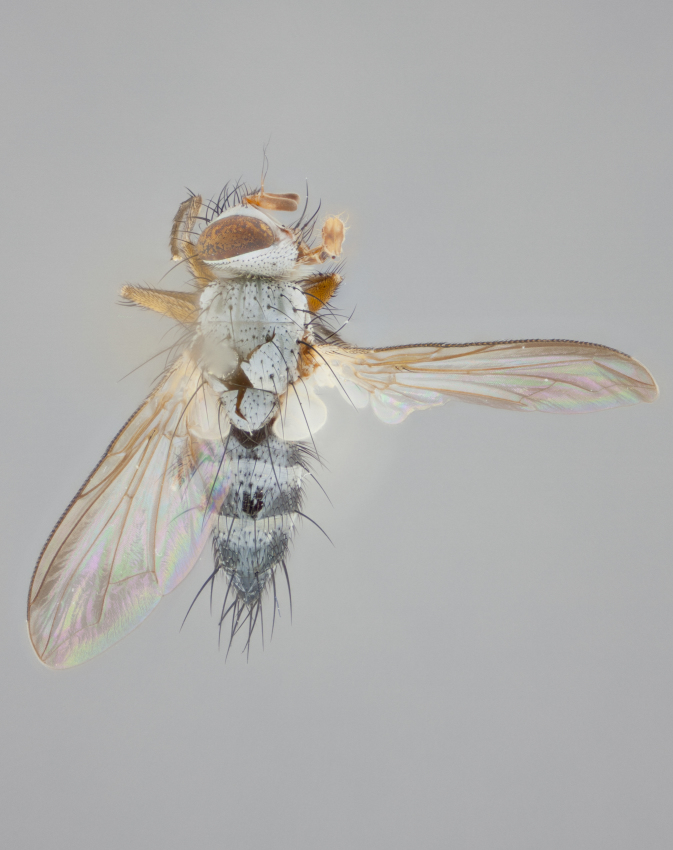
dorsal habitus

**Figure 8b.**
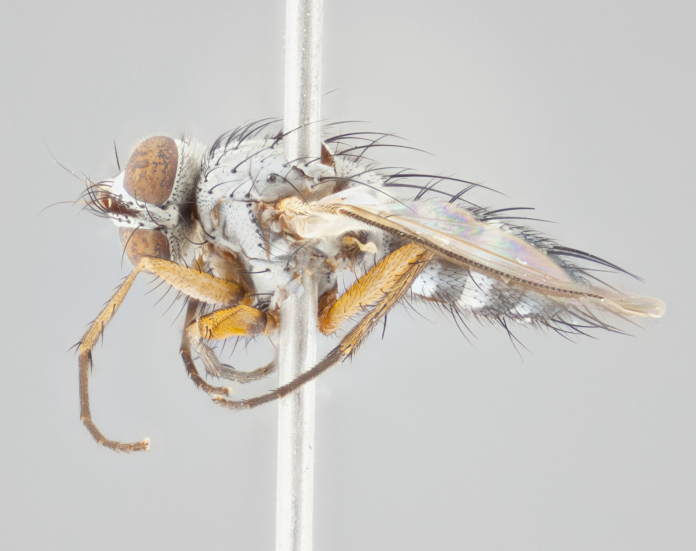
lateral habitus

**Figure 8c.**
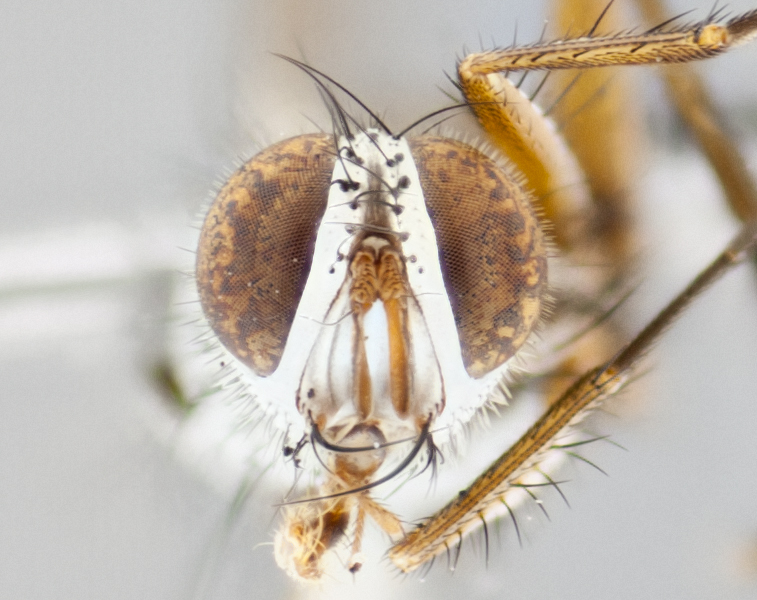
frontal view of head

**Figure 9a.**
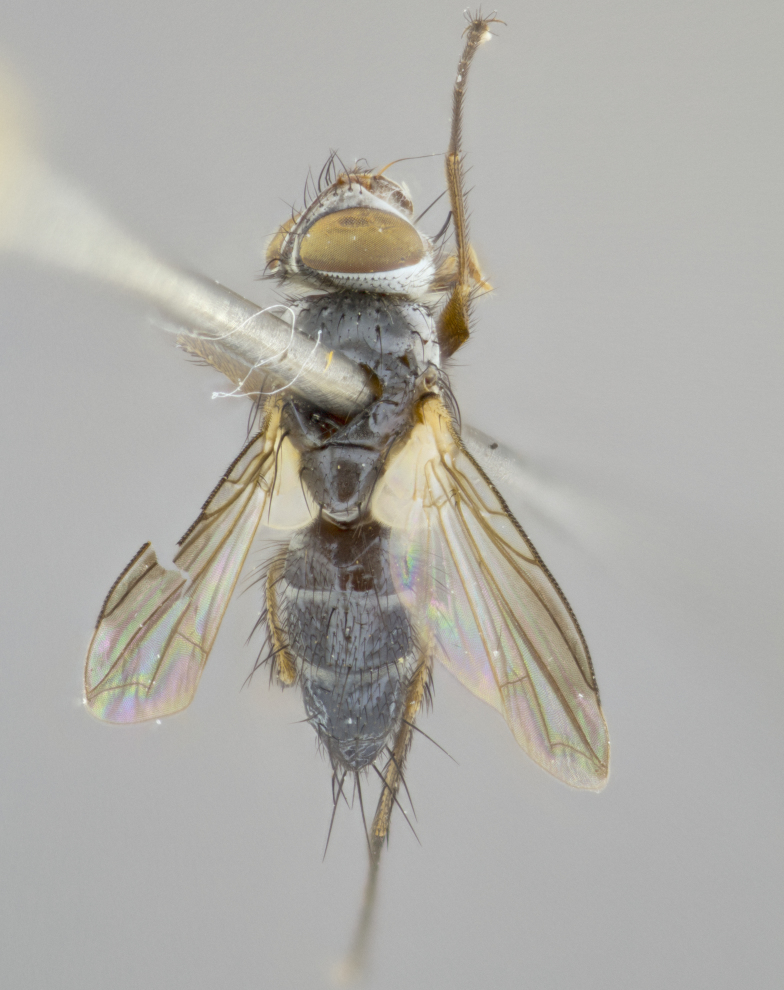
dorsal habitus

**Figure 9b.**
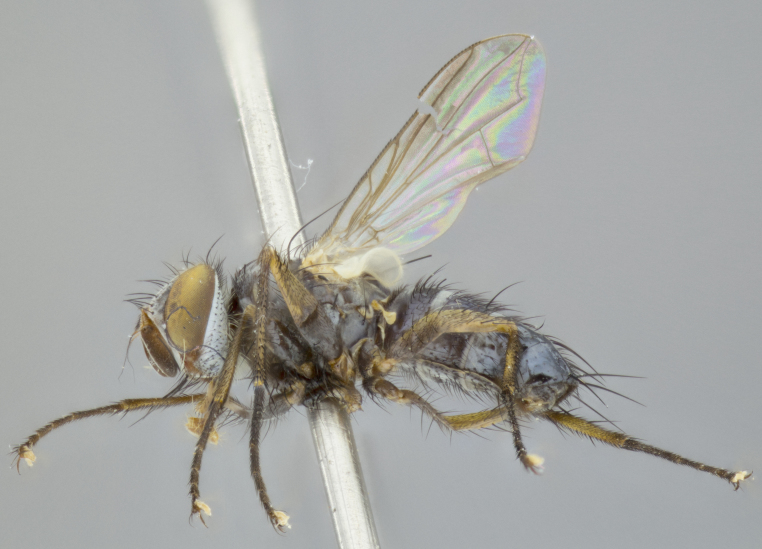
lateral habitus

**Figure 9c.**
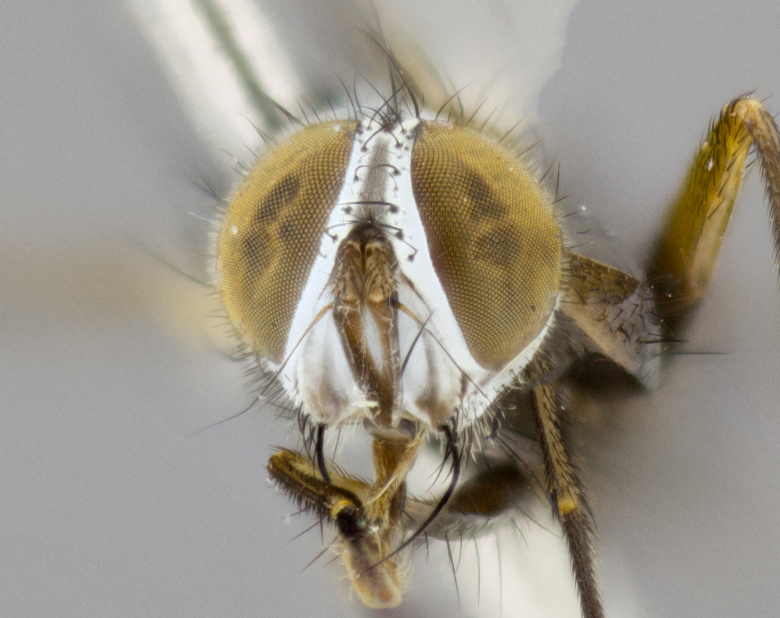
frontal view of head

**Figure 9d.**
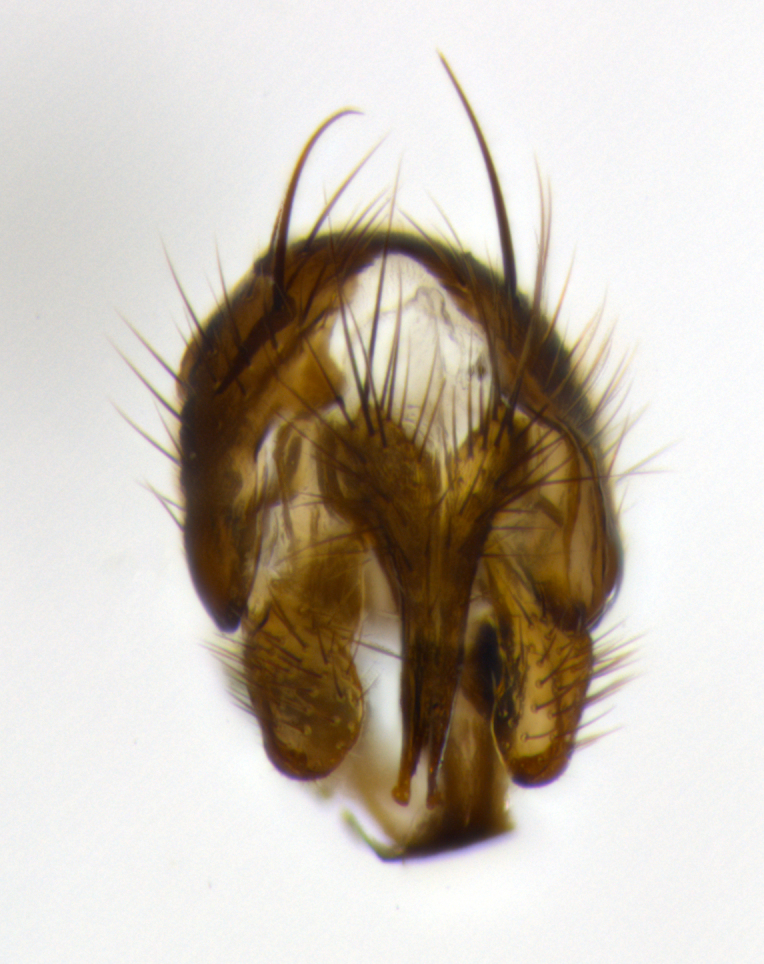
dorsal view of terminalia

**Figure 9e.**
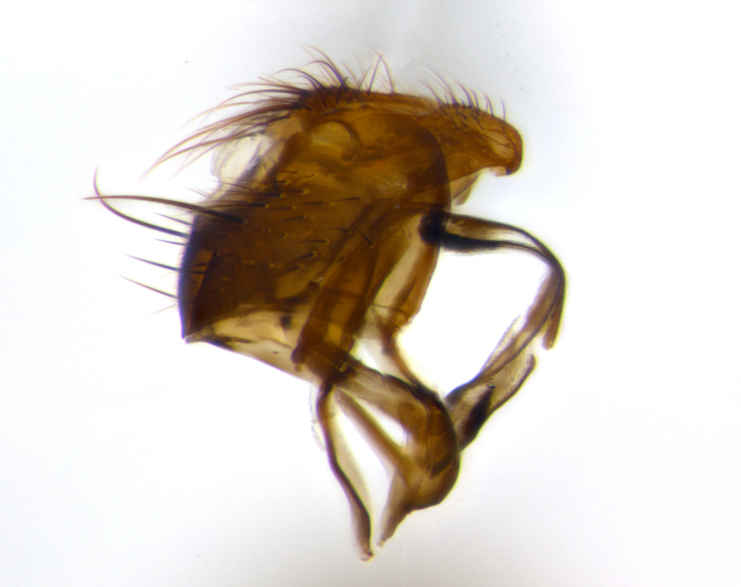
lateral view of terminalia

**Figure 10a.**
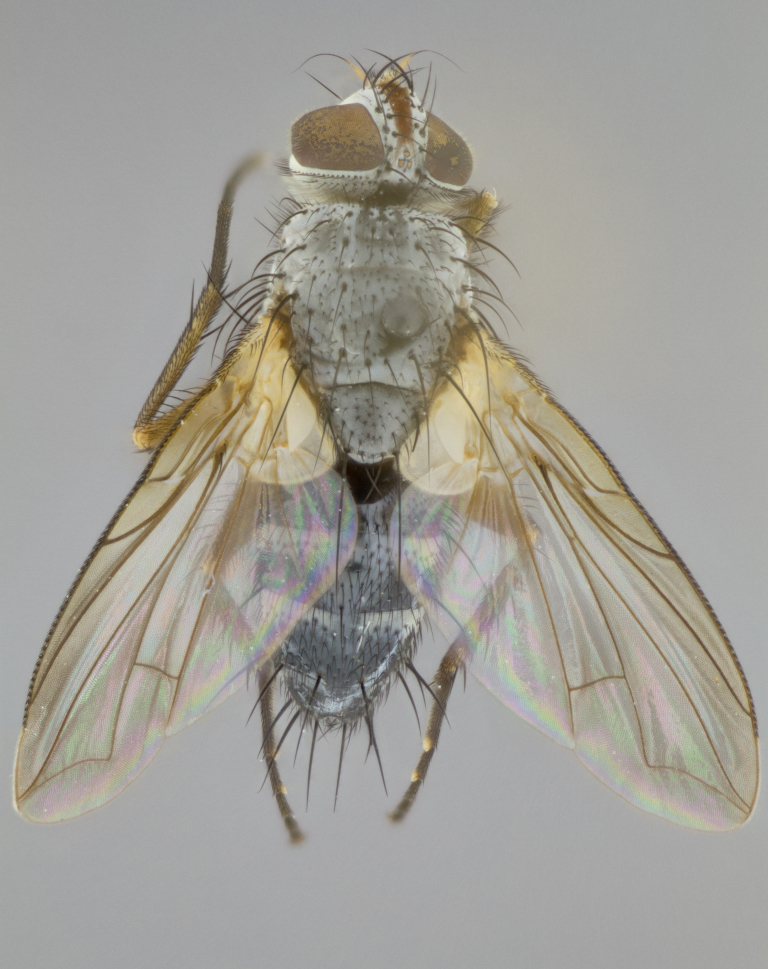
dorsal habitus

**Figure 10b.**
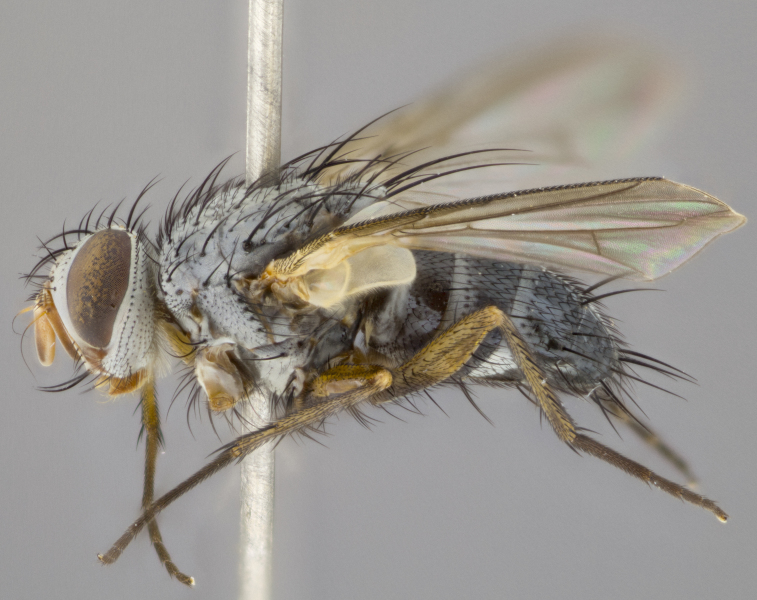
lateral habitus

**Figure 10c.**
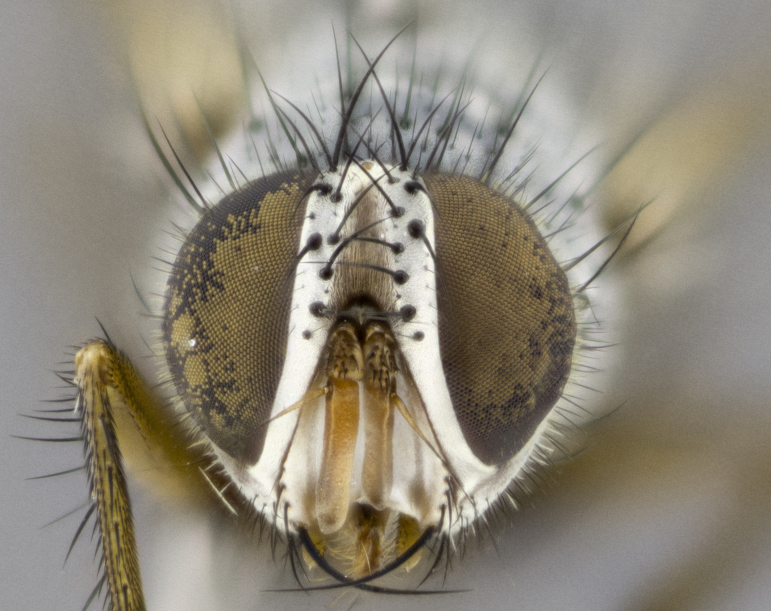
frontal view of head

**Figure 10d.**
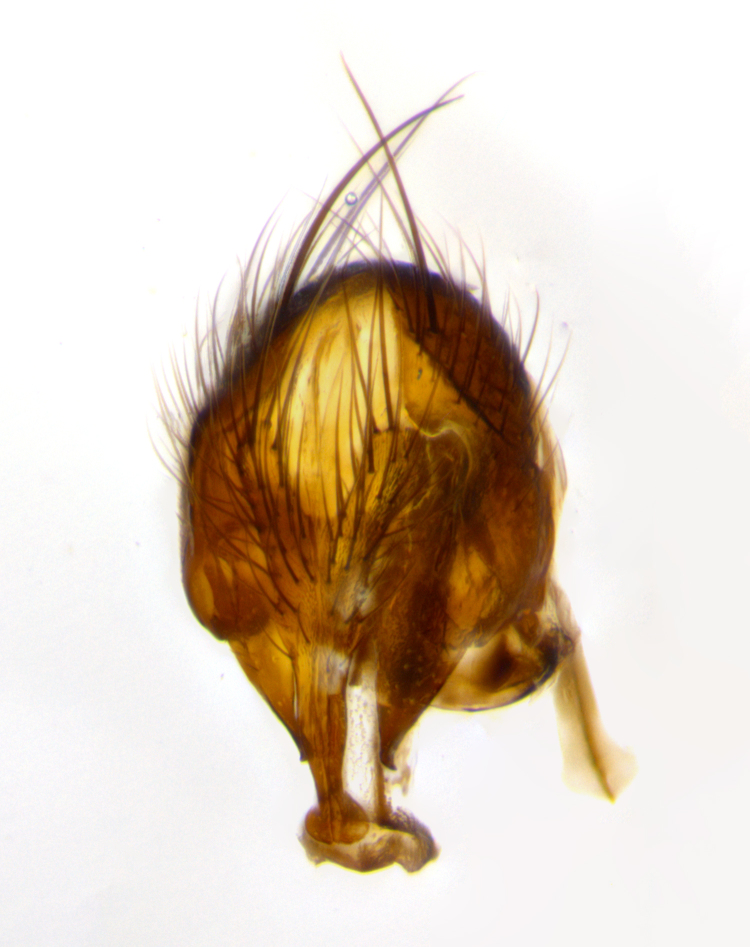
dorsal view of terminalia

**Figure 10e.**
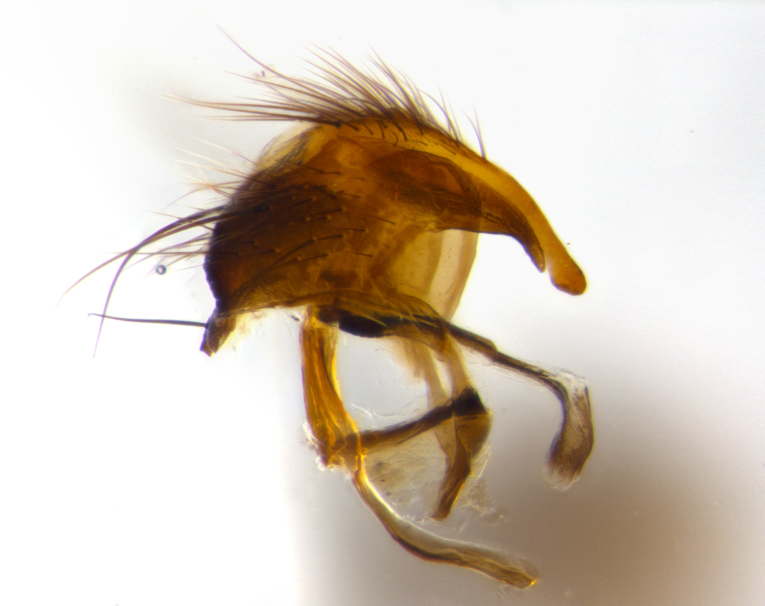
lateral view of terminalia

**Figure 11a.**
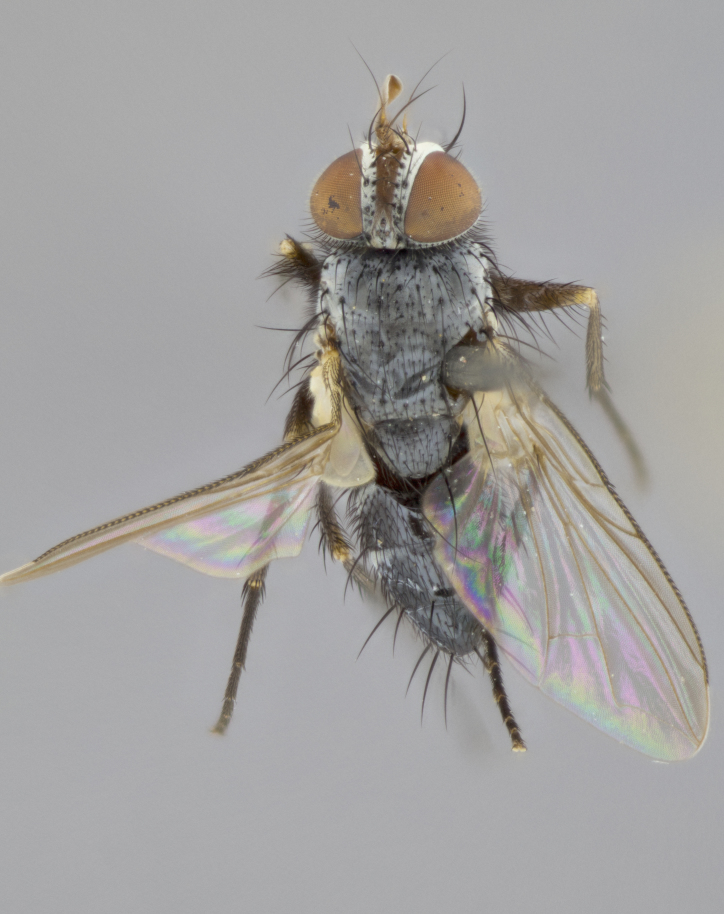
dorsal habitus

**Figure 11b.**
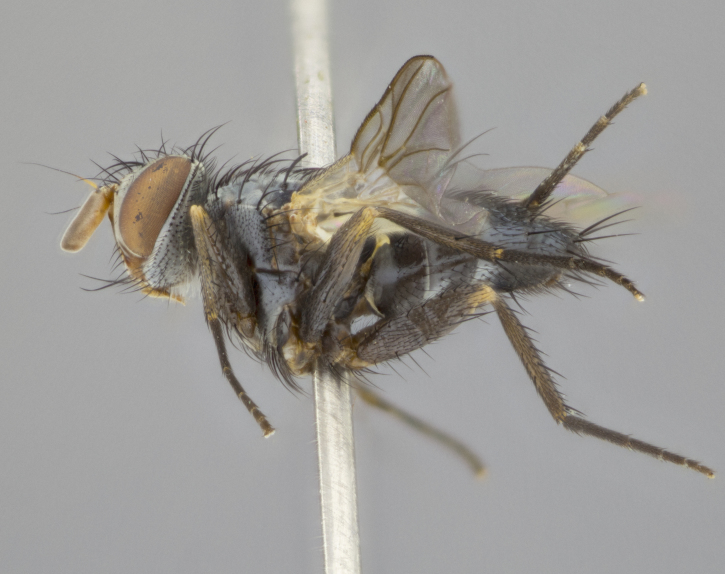
lateral habitus

**Figure 11c.**
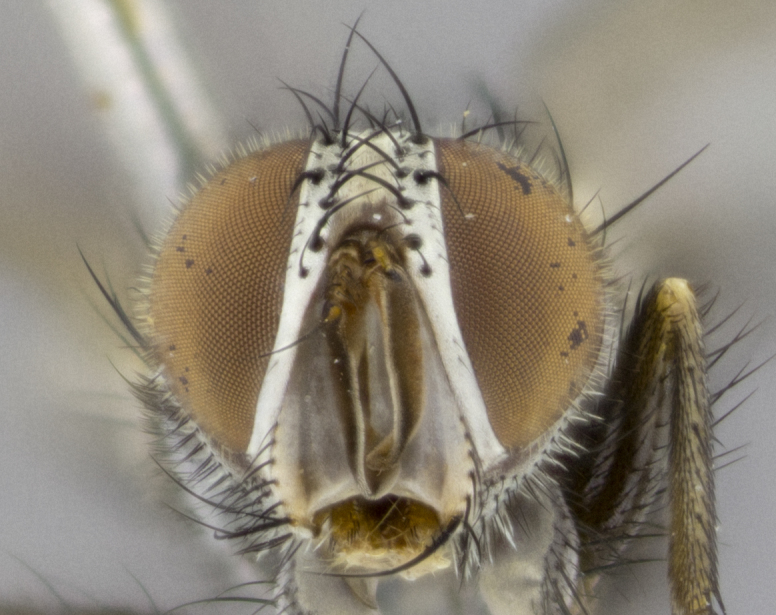
frontal view of head

**Figure 11d.**
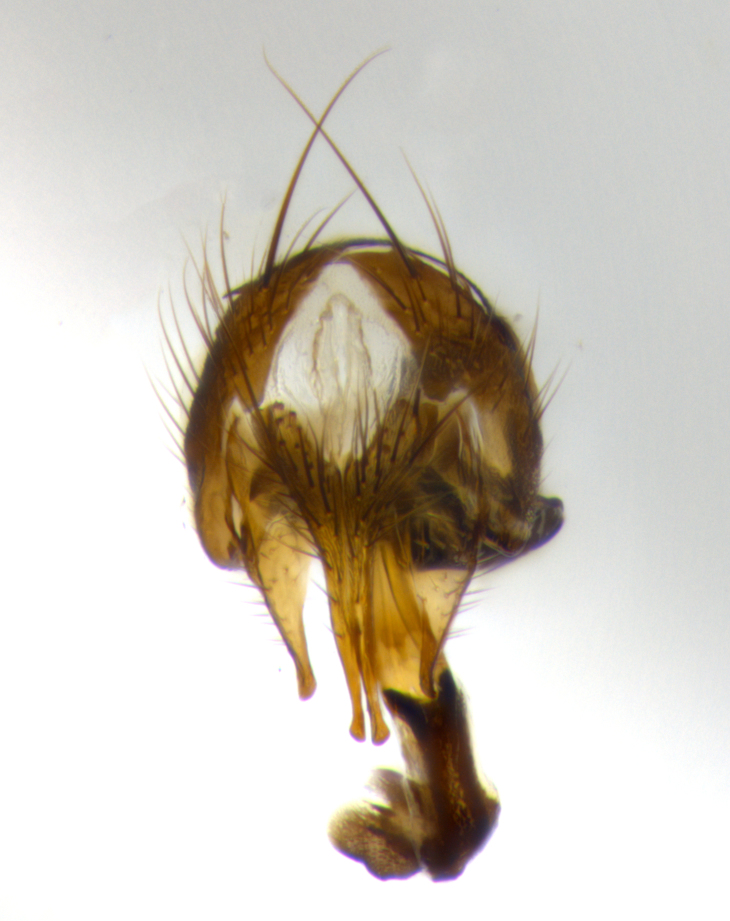
dorsal view of terminalia

**Figure 11e.**
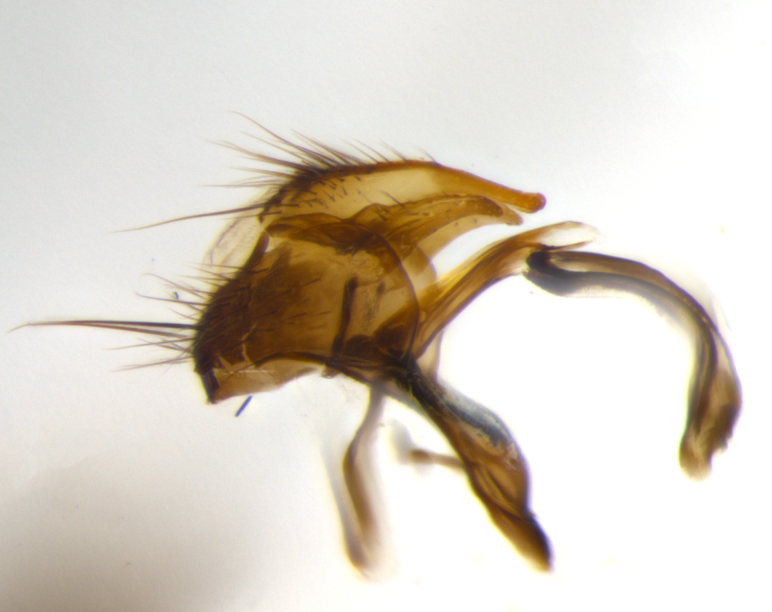
lateral terminalia
