# Supplementary material for: Nine new species of Itaplectops (Diptera: Tachinidae) reared from caterpillars in Area de Conservación Guanacaste, northwestern Costa Rica, with a key to Itaplectops species
Source: Biodivers Data J. 2015 Dec 23;(3):e4596. doi: 10.3897/BDJ.3.e4596 (PMC4698461; doi:10.3897/BDJ.3.e4596)
Supplement: Supplementary material 1 — NJ Tree Itaplectops Nov 2015 [file biodiversity_data_journal-3-e4596-s001.pdf]

BOLD TaxonID Tree

Title : SEARCH: Sample ids(29 ids) [SEARCH3]

Date : 2-November-2015

Data Type : Nucleotide

Distance Model : Kimura 2 Parameter

Marker : COI-5P

Codon Positions : 1st, 2nd, 3rd

Labels : Extra Info, SampleID, Sequence Length

Filters : Length > 200

Colorization : [blue]=Stop Codons [red]=Contamination or misidentification

Sequence Count : 29

Species count : 9

Genus count : 1

Family count : 1

Unidentified : 0

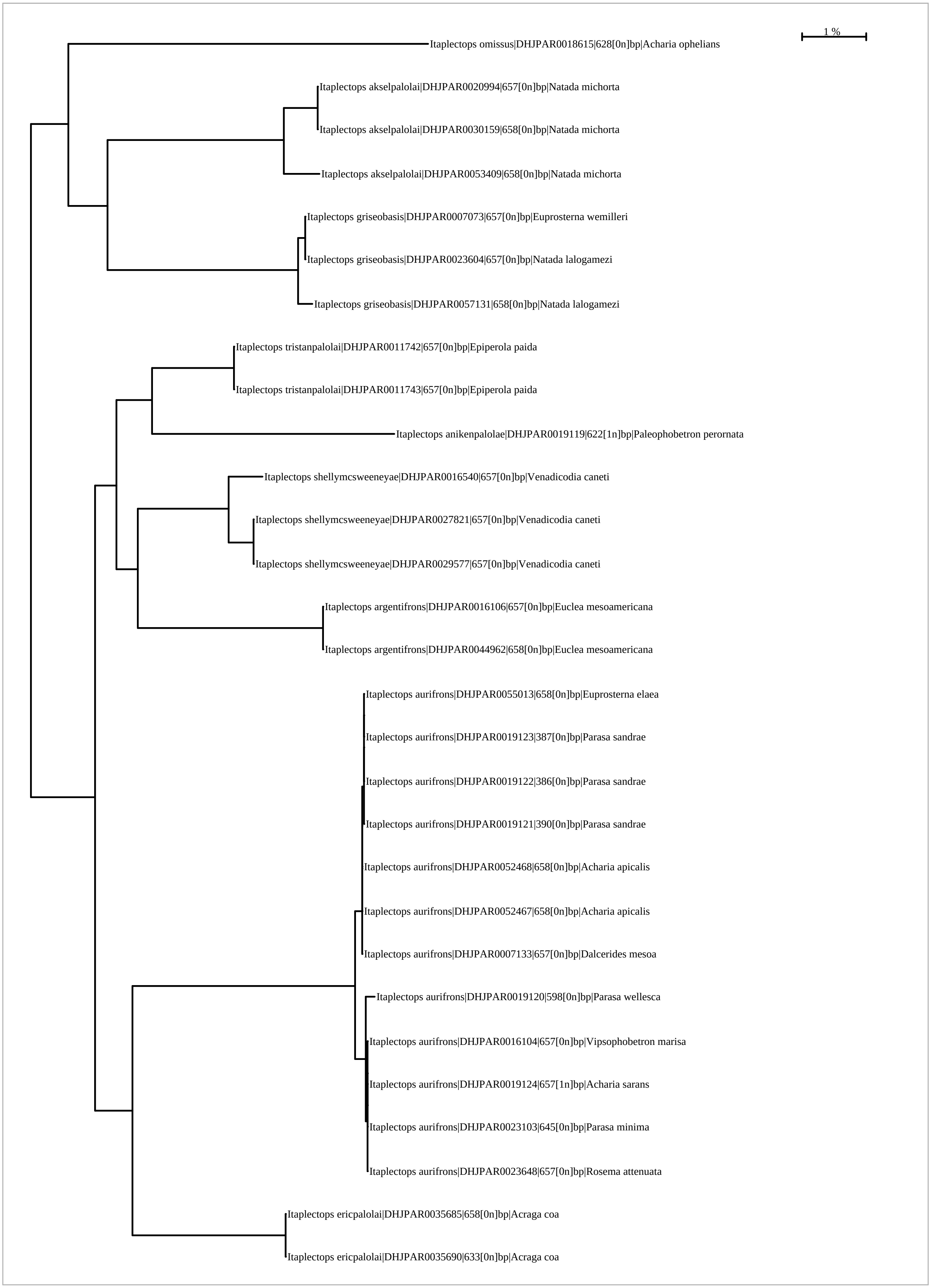
1 %
Itaplectops omissus|DHJPAR0018615|628[0n]bp|Acharia ophelians
Itaplectops akselpalolai|DHJPAR0020994|657[0n]bp|Natada michorta
Itaplectops akselpalolai|DHJPAR0030159|658[0n]bp|Natada michorta
Itaplectops akselpalolai|DHJPAR0053409|658[0n]bp|Natada michorta
Itaplectops griseobasis|DHJPAR0007073|657[0n]bp|Euprosterna wemilleri
Itaplectops griseobasis|DHJPAR0023604|657[0n]bp|Natada lalogamezi
Itaplectops griseobasis|DHJPAR0057131|658[0n]bp|Natada lalogamezi
Itaplectops tristanpalolai|DHJPAR0011742|657[0n]bp|Epiperola paida
Itaplectops tristanpalolai|DHJPAR0011743|657[0n]bp|Epiperola paida
Itaplectops anikenpalolae|DHJPAR0019119|622[1n]bp|Paleophobetron perornata
Itaplectops shellymcsweeneyae|DHJPAR0016540|657[0n]bp|Venadicodia caneti
Itaplectops shellymcsweeneyae|DHJPAR0027821|657[0n]bp|Venadicodia caneti
Itaplectops shellymcsweeneyae|DHJPAR0029577|657[0n]bp|Venadicodia caneti
Itaplectops argentifrons|DHJPAR0016106|657[0n]bp|Euclea mesoamericana
Itaplectops argentifrons|DHJPAR0044962|658[0n]bp|Euclea mesoamericana
Itaplectops aurifrons|DHJPAR0055013|658[0n]bp|Euprosterna elaea
Itaplectops aurifrons|DHJPAR0019123|387[0n]bp|Parasa sandrae
Itaplectops aurifrons|DHJPAR0019122|386[0n]bp|Parasa sandrae
Itaplectops aurifrons|DHJPAR0019121|390[0n]bp|Parasa sandrae
Itaplectops aurifrons|DHJPAR0052468|658[0n]bp|Acharia apicalis
Itaplectops aurifrons|DHJPAR0052467|658[0n]bp|Acharia apicalis
Itaplectops aurifrons|DHJPAR0007133|657[0n]bp|Dalcerides mesoa
Itaplectops aurifrons|DHJPAR0019120|598[0n]bp|Parasa wellesca
Itaplectops aurifrons|DHJPAR0016104|657[0n]bp|Vipsophobetron marisa
Itaplectops aurifrons|DHJPAR0019124|657[1n]bp|Acharia sarans
Itaplectops aurifrons|DHJPAR0023103|645[0n]bp|Parasa minima
Itaplectops aurifrons|DHJPAR0023648|657[0n]bp|Rosema attenuata
Itaplectops ericpalolai|DHJPAR0035685|658[0n]bp|Acraga coa
Itaplectops ericpalolai|DHJPAR0035690|633[0n]bp|Acraga coa
